# Developing a clinical–environmental–genotypic prognostic index for relapsing-onset multiple sclerosis and clinically isolated syndrome

**DOI:** 10.1093/braincomms/fcab288

**Published:** 2021-12-04

**Authors:** Valery Fuh-Ngwa, Yuan Zhou, Jac C Charlesworth, Anne-Louise Ponsonby, Steve Simpson-Yap, Jeannette Lechner-Scott, Bruce V Taylor, AusLong/Ausimmune Investigators Group, Keith Dear, Terry Dwyer, Ingrid van der Mei, Trevor Kilpatrick, David Williams, Jeanette Lechner-Scott, Cameron Shaw, Caron Chapman, Alan Coulthard, Michael P Pender, Patricia Valery

**Affiliations:** 1 Menzies Institute for Medical Research, University of Tasmania, Hobart, TAS, 7000, Australia; 2 Developing Brain Division, The Florey Institute for Neuroscience and Mental Health, University of Melbourne Murdoch Children’s Research Institute, Royal Children’s Hospital, Parkville, VIC, 3052, Australia; 3 Neuroepidemiology Unit, Melbourne School of Population & Global Health, The University of Melbourne, Melbourne, VIC, 3053, Australia; 4 Department of Neurology, Hunter Medical Research Institute, University of Newcastle, Callaghan, NSW, 2310, Australia; 5 Department of Neurology, John Hunter Hospital, Newcastle, NSW, 2310, Australia

**Keywords:** multiple sclerosis, prognostic index, clinical–environmental, genetic variants, dynamic predictions

## Abstract

Our inability to reliably predict disease outcomes in multiple sclerosis remains an issue for clinicians and clinical trialists. This study aims to create, from available clinical, genetic and environmental factors; a clinical–environmental–genotypic prognostic index to predict the probability of new relapses and disability worsening. The analyses cohort included prospectively assessed multiple sclerosis cases (*N* = 253) with 2858 repeated observations measured over 10 years. *N* = 219 had been diagnosed as relapsing-onset, while *N* = 34 remained as clinically isolated syndrome by the 10th-year review. Genotype data were available for 199 genetic variants associated with multiple sclerosis risk. Penalized Cox regression models were used to select potential genetic variants and predict risk for relapses and/or worsening of disability. Multivariable Cox regression models with backward elimination were then used to construct clinical–environmental, genetic and clinical–environmental–genotypic prognostic index, respectively. Robust time-course predictions were obtained by Landmarking. To validate our models, Weibull calibration models were used, and the Chi-square statistics, Harrell’s C-index and *pseudo*-*R*^2^ were used to compare models. The predictive performance at diagnosis was evaluated using the Kullback–Leibler and Brier (dynamic) prediction error (reduction) curves. The combined index (clinical–environmental–genotypic) predicted a quadratic time-dynamic disease course in terms of worsening (HR = 2.74, CI: 2.00–3.76; *pseudo*-*R*^2^=0.64; C-index = 0.76), relapses (HR = 2.16, CI: 1.74–2.68; *pseudo*-*R*^2^ = 0.91; C-index = 0.85), or both (HR = 3.32, CI: 1.88–5.86; *pseudo*-*R*^2^ = 0.72; C-index = 0.77). The Kullback–Leibler and Brier curves suggested that for short-term prognosis (≤5 years from diagnosis), the clinical–environmental components of disease were more relevant, whereas the genetic components reduced the prediction errors only in the long-term (≥5 years from diagnosis). The combined components performed slightly better than the individual ones, although their prognostic sensitivities were largely modulated by the clinical–environmental components. We have created a clinical–environmental–genotypic prognostic index using relevant clinical, environmental, and genetic predictors, and obtained robust dynamic predictions for the probability of developing new relapses and worsening of symptoms in multiple sclerosis. Our prognostic index provides reliable information that is relevant for long-term prognostication and may be used as a selection criterion and risk stratification tool for clinical trials. Further work to investigate component interactions is required and to validate the index in independent data sets.

## Teaser

Utilizing clinical, environmental and genetic data, Fuh-Ngwa et al. developed a prognostic index capable of predicting the complex disease dynamics (relapses and worsening of disability) in relapsing-onset Multiple Sclerosis. Using their index, the authors predicted a quadratic time-dynamic disease course in terms of developing relapses and worsening of disability long-term.

## Graphical Abstract

**Graphical Abstract:**
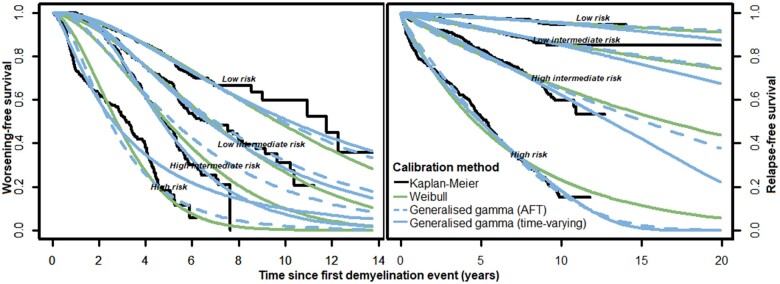

## Introduction

Our inability to reliably predict the course of disease progression in the short and long-term in people with relapsing onset multiple sclerosis (ROMS) and/or clinically isolated syndrome (CIS) remains a significant issue for the MS community. Despite significant progress in understanding the pathophysiology of MS, the disease course remains largely unpredictable,^1^ with considerable inter and intra-individual variation.^2–5^ The limitation of current predictors for prognostication is exemplified by conventional brain MRI. For instance, MRI lesion measures are currently incorporated in established criteria for the MS diagnosis but have limited predictive values for disease severity.^1^^,^^6^

Currently, a prognostic index incorporating reported clinical^7–11^; environmental^12–17^; and genetic factors^18–20^; and capable of discriminating potential disease course at a first demyelinating event (FDE) or at the time of MS diagnosis is not available. Mandrioli et al.^21^ developed a multifactorial prognostic index for MS that incorporated only cerebrospinal fluid parameters while adjusting for baseline clinical and demographics factors, but leaving out genetic and environmental components. Perhaps the low variability ($r^{2}=21.4\%$) captured in the more recent genetic model of MS disease severity^22^ could be attributed to the missing clinical and environmental components that play a major role in MS disease severity as reported elsewhere.^23^

We had previously investigated the role of genetic susceptibility variants using the standard time-invariant genetic risk scores estimated from SNPs that were predictive of clinical course in MS^24^ but were unable to include important clinical and environmental information. However, whether combining genotype information with clinical and environmental information can improve predictive performance and increase the variation explained has not been studied. Furthermore, how these variables are combined may be important in terms of overall prediction accuracy.

Nevertheless, statistical learning methods that combine the clinical and environmental factors with the genetic information (e.g. the ‘super learner’ of van Houwelingen and Putter^25^ and van der Laan et al.^26^) into a prognostic index have been shown to improve predictive performance in terms of risk stratification. Moreover, allowing the effects of the genetic variants to vary with time or landmarking their effects in the prognostic index may provide useful biological information that could be missed otherwise.^27^^,^^28^ That is the prognostic indices may be time-dependent such that factors that predict subsequent prognosis may differ depending on the disease duration.

This study aimed to create, from prospectively collected clinical, environmental and genotype data, a clinical–environmental–genotypic (clinical–env–genotypic, hereinafter) prognostic index (CEGPI) predicting the probability of developing new relapses and disability worsening outcomes from FDE. We also aimed to obtain robust dynamic estimates from FDE and 5 years post-onset, of the risk for relapses and worsening of disability, using a landmark approach. By utilizing data from persons from the time of a first clinical diagnosis of CNS demyelination (those who subsequently developed ROMS and/or remained as CIS up to the 10th year post-onset), we hypothesized that both clinical, environmental, and genetic factors in combination and singly, would predict metrics of disease severity. We also hypothesized that these factors would be time dynamic and thus knowledge of disease duration will be an important driver of disease progression and prognostication.

## Materials and methods

### Data and study design

Data were derived from the Ausimmune Longitudinal (AusLong) study.^29^ The Auslong study is a population-based prospective cohort study of FDE participants recruited soon after their referral episode. All Participant samples were genotyped using the Illumina MS Chip,^30^ which includes ∼240 000 exome SNPs based on the Human Exome-12 v1.2 array plus an additional ∼88 600 MS-relevant variants added as a customized component. These data were imputed to ∼2.9 million SNPs using the algorithm implemented in Minimac 3^31^ using the 1000-genome phase-3^32^ as the reference panel. SNP genotypes were captured for 199 of the 233 MS risk SNPs published by the International MS Genetics Consortium.^33^ The analysis cohort included 253 participants with 2858 repeated observations measured over 10 years. Of this, 219 had been diagnosed with ROMS, while 34 remained as CIS by the 10th year review.

### Definition of outcome measures

The study outcomes considered were:

- The time to relapse and/or recurrence of relapsing events (RRE); where relapse was defined according to the 2017 McDonald criteria.^34^
- The time to change in the level of the Expanded Disability Status Scale (EDSS): Here, the follow-up measurements for each individual included the clinical status ‘*worsening*’ versus ‘*not-worsening*’, and the outcome denoted as ‘WoD’ (worsening of disability) hereafter.
- The time to ‘relapse and/or worsening of disability,’ denoted ‘RwoD’ hereafter. This is a combination of both the RRE and the WoD status.

We restructured the data based on the Markov assumptions of a continuous-time evolution of MS disease course (EDSS transition),^35–37^ whereas the definition of WoD (‘*worsening*’ versus ‘*not-worsening*’) stems from previous studies.^2–5^ By restructuring the data, and defining the WoD measure, we preserved the natural interpretation of MS progression in terms of stage progression, meanwhile ensuring the *intra-* and *inter-ratter* variability are assessed evenly across the entire scale.

### Statistical analysis

#### Selection of potential genetic predictors

Sample quality control of the genotype data was performed as described in Anderson et al.^38^ To predict time to RRE, WoD and RWoD, a *global test* for the added prognostic value of all SNPs (*n* = 199) that passed the quality control stage was done using the Goeman’s ‘globaltest′ R-package.^39^ Here, we test the null hypothesis of no additional prognostic value of the genetic markers given the clinical and environmental predictors (clinical–env, hereinafter). Following this, we applied a least absolute shrinkage and a selection operator (LASSO) within the framework of survival models (Cox-LASSO) with leave-one-out cross-validation (LOOCV) to select potential SNPs using Goeman’s ‘penalized’ R-package.^40^ The Cox-LASSO regression was adopted given its accepted good performance, inherent variable selection routine, and the ability to accommodate correlated SNPs and event times.^25^^,^^41^ Unbiased estimates for significant SNPs with non-zero effect sizes were obtained using the ‘backfitting’ algorithm of Sauerbrei and Royston.^42^

For each survival endpoint that we analysed, an additive genetic model was assumed, and the significance level to stay in the model was set to P≤0.05. The effects of the resulting SNPs were allowed to be landmark-dependent and/or vary with the logarithm of the inter-attack intervals (i.e. the difference between event start and event stop times) for each endpoint. Note that the SNPs included were those suggestive of MS risk according to the international MS genetic consortium.^43^ After quality control, 17-HLA SNPs from the major histocompatibility complex (MHC) region and 182 non-MHC autosomal SNPs formed the basis for initial selection with Cox-LASSO. The final genetic models for each survival endpoint included the effects of the primary signal that maps to the *HLA-DRB1* gene (HLA-DRB1*15:01 allele; RefSNP: *rs3129889*) following its previously established primary role in MS susceptibility.^44^ To be specific, we allowed the effects of the SNPs to be landmark-dependent and/or vary with the logarithm of the inter-attack intervals, as well as interactions with standardized latitudinal coordinates to adjust for gene–environment (GxE) interactions following previous findings.^23^

#### Selection of potential clinical and environmental predictors

The predictive significance of multiple clinical–env predictors of MS risk and/or disease time-course were assessed including; age at FDE, body mass index (BMI), sex, relapse counts, the intervals between attacks, baseline 25-hydroxy vitamin D levels [25(OH)D], smoking status (tobacco or marijuana), latitude, T2 lesion counts on baseline MRI (T2L), hours of sunlight exposure, duration of disease-modifying therapies (DMTs) and vitamin D supplementation; were investigated using multivariate survival models. Initially, we fitted crude Cox models which included additional predictors such as recent immunization status (those who have had any immunization done since their last review), change in job status, hospital anxiety depression scores (HADS), study site, income levels and employment status; to gain insight into the prognostic effect for each factor. Core clinical–env models for each survival endpoint were then constructed using backward selection (P≤0.05) with a systematic search for multifactorial polynomial terms according to Sauerbrei and Royston.^42^ These clinical–env predictors were carefully selected from those reported in previous studies (Supplementary Table 1) that examined their roles in MS risk and disease progression. Regardless of statistical significance in each survival endpoint; age at FDE, sex, study site, duration of DMTs and T2L, were included as possible adjustments in the core clinical–env models based on their relevance in MS.^9^^,^^10^^,^^13^^,^^17^^,^^21^^,^^45–49^

#### Synthesis of the prognostic index

Using the approach of van Houwelingen and Putter^25^ with LOOCV to avoid model overfitting, we created:

1. A Clinical–Env Prognostic Index (CEPI), from a multivariable Cox regression analysis using the core clinical-env predictors that passed the selection stage;
2. A Genetic Prognostic Index (GPI), from a multivariable Cox regression on SNPs that passed the selection stage; and
3. A Clinical–Env–Genotypic Prognostic Index (CEGPI), from a linear combination of CEPI + GPI, after performing a supermodel Cox regression analysis on CEPI and GPI, respectively.

In (3), the CEGPI is constructed as $\mathrm{CEGPI}=\alpha_{1}*\mathrm{CEPI}+\;\alpha_{2}*\mathrm{GPI}$, where $\alpha_{1}(\alpha_{2})$ are, respectively, the log-hazard ratios for CEPI and GPI. To avoid potential violations of the Cox proportional hazard assumption induced by time-dependent covariates (Supplementary Table 1), the Anderson–Gill (AG) model^50^ was used to obtained robust standard errors (SE) (sandwich variance estimates) from which robust confidence intervals (CIs) were estimated. Next, we stratified the clinical–env and genetic supermodels by EDSS to in other to capture the entire disease state space^51^. By so doing, the inter and intra-individual variability is captured evenly along the entire scale of EDSS.^36^^,^^51^ We also stratified by the dynamic conversion status (CDMS, clinically defined MS) to account for differences that exist in our study population (different baseline hazards for ROMS & CIS). Right-censored events times were assumed for each survival models that we fitted.

#### Dynamic prediction using the prognostic index

Using the obtained indices, we performed a supermodel Cox regression and constructed a ‘super learner’ from which dynamic predictions were achieved by Landmarking described elsewhere.^25^^,^^28^ By definition, a supermodel Cox regression is that which is executed on the resulting cross-validated prognostic indices (i.e. on CEPI only, or GPI only, or CEPI and GPI); whereas a ‘super learner’ is a supermodel Cox regression on CEGPI only. Robust estimates (averaged over five landmark data sets created at time points $t_{Lm}$ = 0, 1,2, 3, 4 and 5 years, with a prediction window of width w= 5 years) for the log-hazards were then obtained by fitting proportional baseline and stratified landmark supermodels, allowing for linear and quadratic interactions with the landmark times.

#### Validation of the prognostic index

To validate the obtained indices, the *validation by calibration* approach of van Houwelingen^52^ was adopted, wherein Weibull calibration models were fitted on four risk groups defined as: ‘$\mathrm{IP}I_{1}$’ = low risk (0–25% risk); ‘$\mathrm{IP}I_{2}’$ = low intermediate risk (25–50% risk); ′$\mathrm{IP}I_{3}$’ = high intermediate risk (50–75%) and ′$\mathrm{IP}I_{4}$’ = high risk (75–100%) as in van Houwelingen.^52^ We adjusted the baseline hazards in our data using information from two populations namely: the British Columbia cohort,^53^ and the Phase III Tysabri trial from North America.^54^ These populations were chosen due to their large sample sizes. Finally, the model-$\chi^{2}$, Harrell’s C-index and *pseudo*-$R^{2}$ were used for model comparison, while the overall performance of the index at diagnosis was evaluated using the Kullback–Leibler and Brier (dynamic) prediction error (reduction) curves. All statistical analyses were performed using the R-software version 3.6.0.

#### Estimating risk scores for disease progression

The prognostic model used for obtaining the risk scores for each IPI subgroup of the CEGPI is given by

(1) $$h\left(t|{\mathrm{IPI}}_{\mathrm{CEGPI}}\right)=h_{0}\left(t\right)\times \text{exp}(\gamma_{1}*IPI_{2}+\;\gamma_{2}*IPI_{3}+\gamma_{3}*IPI_{4})$$

where $h\left(t|{\mathrm{IPI}}_{\mathrm{CEGPI}}\right)$ is the hazard of a relapse or worsening event at any given time *t*, $h_{0}\left(t\right)$ is the baseline hazard, and $\gamma_{\left(.\right)}$ are the regression effects of the prognostic subgroups. The probability, at given time, of having a worsening or relapsing event, given all available genetic and clinical–env components of disease; and conditional on the IPI subgroups of the CEGPI, was obtained from the prognostic formula (1) using:

(2) $$\lambda_{\mathrm{IPI}}=\frac{HR_{\mathrm{IPI}}}{1+HR_{\mathrm{IPI}}}$$

where $\lambda_{\mathrm{IPI}}$ is the risk score of the prognostic subgroup, and $HR_{\mathrm{IPI}}=\exp(\gamma_{\left(.\right)})$ is the hazard ratio. The risk scores range between 0 and 1, denoting the probability at given time, of observing an EDSS score that is greater than or equal to the previous score.

### Data availability

The Ausimmune/AusLong data used to construct the CEGPIs are available from the authors upon reasonable request. The data are not publicly available due to privacy and ethical restrictions.

## Results

In our analysis cohort, 77.5% (*n* = 196) were females, and the mean age at study entry was 36.6 years (SD = 9.2). The mean times to relapse were 10.67 months (SD = 6.00) for males and 10.31 months (SD = 6.10) for females. The annual relapse rates were 0.23 ($N_{\mathrm{events}}$=84, SD = 1.42, range = 0–7) in males and 1.35 ($N_{\mathrm{events}}$=493, SD = 2.91, range = 0–25) in females; where $N_{\mathrm{events}}$ is the total number of post onset relapses. The mean times until a change in the EDSS level were 7.00 months (SD=5.30) and 7.30 months (SD = 4.80) for males and females, respectively. The 5 and 10 years cohort characteristics are given on Supplementary Table 1.

### Clinical–env prognostic factors

Table 1 shows the results for the core models, while Supplementary Table 2 shows the results from the crude models. From Table 1, seven predictors (T2L, BMI, relapse counts, recent immunization status, HADS, seasonal changes in hours of sunlight exposure, and income levels significantly increased the risk for WoD annually after adjusting for risk factors, such as sex, age at FDE, DDMTs and study site. Vitamin D supplementation and shorter inter-attack intervals reduced the likelihood of worsening each year. Although baseline 25(OH)D was minimally protective, its effect on disability worsening was not significant. Except for recent immunization status and DDMTs, the above-mentioned factors were also predictive of relapse risk. In addition to these, a ‘*worsening*’ clinical status is an important driver of relapse risk, but importantly the reverse was not supported when predicting the risk for worsening. From the core models (Table 1), similar clinical–env factors were included to predict each endpoint. With the exception of age at FDE, sex and study site, the direction of the remaining effects across the endpoints were consistent.

**Table 1:** Regression coefficients (β), standard errors (SE) and *P*-values (*P*) for the candidate clinical and environmental predictors included in the clinical–environmental prognostic index (CEPI) when predicting the risk of worsening of disease (WoD), relapses (RRE) and relapse and/or worsening of disease (RWoD). Estimates for clinical predictors not included in the final models are left blank as they did not pass the significance level (α≤0.05) to stay in the model

		**Worsening of disease** N= 2858; D=1011			**Relapses** N=2858; D=564			**Relapse and/or worsening of disease** N=2858; D=1377		
Clinical variables	Categories	β	SE	*P*	β	SE	*P*	β	SE	*P*
Baseline predictors										
* *Age at FDE (years)		0.01	0.01	0.07	−0.02	0.01	<0.01	<−0.01	<0.01	0.86
* *Sex	Female	−0.06	0.09	0.55	0.02	0.11	0.88	−0.10	0.08	0.22
* *Study site	TAS	0.09	0.13	0.52	−0.04	0.12	0.71	−0.03	0.09	0.71
	VIC	−0.08	0.15	0.57	0.11	0.11	0.33	−0.14	0.10	0.17
	NSW	0.02	0.16	0.92	0.20	0.11	0.07	0.11	0.10	0.27
	QLD	Reference			Reference			Reference		
* *25(OH)D (nmol/l)		<−0.01	<0.01	0.12	<−0.01	<0.01	0.83	<−0.01	<0.01	<0.01
* *Smoke tobacco	Yes	–	–	–	–	–	–	–	–	–
* *Smoke marijuana	Yes	–	–	–	–	–	–	–	–	–
* *Educational level	HE	–	–	–	–	–	–	–	–	–
	SE	−0.15	0.10	0.13				−0.12	0.07	0.06
	LSE	Reference			Reference			Reference		
* *Number of T2 lesions		0.45	0.09	<0.01	0.25	0.06	<0.01	0.26	0.06	<0.01
* *Duration of DMT		−0.01	0.19	0.94	−0.22	0.14	0.12	−0.01	0.06	0.85
Time-dependent predictors										
* *RRE	Yes	–	–	–	–	–	–	–	–	–
* *WoD	Yes	–	–	–	0.25	0.11	0.02	–	–	–
* *Body mass index (kg/m^2^)		1.42	0.41	<0.01	0.57	0.32	0.07	2.51	0.99	0.01
* *Relapse counts		0.42	0.08	<0.01	0.29	0.07	<0.01	0.67	0.05	<0.01
* *Recent immunization Yes		0.29	0.09	0.01	−0.12	0.14	0.38	0.09	0.08	0.23
* *Vitamin D supplements	Yes	−0.69	0.19	<0.01	−3.87	0.17	<0.01	−0.61	0.18	<0.01
* *HADS		0.28	0.09	<0.01	0.44	0.13	<0.01	0.60	0.07	<0.01
* *Job change	Yes	–	–	–	–	–	–	–	–	–
* *Employment status	FT	–	–	–	–	–	–	−0.27	0.10	0.01
	DP	−0.25	0.17	0.15	–	–	–	−0.30	0.13	0.02
	PT/WH	–	–	–	–	–	–	–	–	–
	UE	Reference			Reference			Reference		
Δ in sunlight exposure (h)		0.07	0.03	0.01	–	–	–	0.07	0.03	0.01
* *Income levels	$1500–$2000	–	–	–	0.51	0.16	<0.01	0.45	0.17	0.01
	$600–$1499	0.58	0.14	<0.01	0.84	0.13	<0.01	0.64	0.12	<0.01
	$1–$599	0.38	0.15	0.01	0.52	0.15	<0.01	0.46	0.12	<0.01
	$0	Reference			Reference			Reference		
* *Log(Inter-attack intervals)		−0.24	0.02	<0.01	−0.99	0.05	<0.01	−3.59	0.21	<0.01

NB: The actual values for *P*-values < 0.01 range between $10^{-3}\;\mathrm{and}\;10^{-12}$.

D= number of events, N=number of observations.

DP, disability pension; FT, full time; HADS, hospital anxiety depression score; HE, higher education; LSE, less than secondary education; PT/WH, part-time/work from home; SE, secondary education; UE, unemployed.

NSW, New South Wales; QLD, Queensland; TAS, Tasmania; VIC, Victoria.

Inter-attack intervals = Difference between event stop and event start time.

Δ in sunlight exposure: Difference in hours of sunlight exposure (winter-summer).

FDE, first demyelinating event; 25(OH)D, 25 hydroxy vitamin D levels measured in units of nmol/l.

### Genetic prognostic factors

The global test for the null hypothesis of no additional prognostic values of all 199 SNPs markers given the clinical–env predictors was rejected for each survival endpoint. In particular, we estimated the global statistics as:$\;Z_{\mathrm{WoD}}^{*}$ = 0.60, $Z_{\mathrm{RRE}}^{*}$ = 2.07, $Z_{\mathrm{RWoD}}^{*}$ = 0.60; and the *P*-values as $P_{\mathrm{WoD}}$ = 1.2 × 10^−5^, $P_{\mathrm{RRE}}$ = 1.8 × 10^−162^, $P_{\mathrm{RWoD}}$ = 2.8 × 10^−11^, when evaluating the predictive significance for the risk of WoD, RRE and RWoD, respectively. Unbiased estimates for the SNPs included in the core genetic models are shown on Supplementary Table 3. Noteworthy is the significant time-dynamic (*) and the latitudinal (ǂ) effects for some SNPs on the endpoints. Although the effects of such SNPs decreased with time, they remained strong. Regarding the effects of HLA-DRB1*15:01 we observed a non-significant main effect in terms of WoD (HR = 0.90; *P* = 0.79), RRE (HR = 1.19, *P* = 0.78) and RWoD (HR = 2.94, *P* = 0.34). Also, its interaction with time and standardized latitudinal coordinates were not significant in the WoD and RRE endpoints. However, after adjustment for relapses (RWoD), its effect on worsening events increased significantly with time (HR = 1.17; *P* = 0.01). Given the core clinical–env predictors, the test for predictive significance indicated that the genetic variants have additional prognostic value for disease time-course prediction.

### Distribution of the prognostic indices

**Figure 1:**
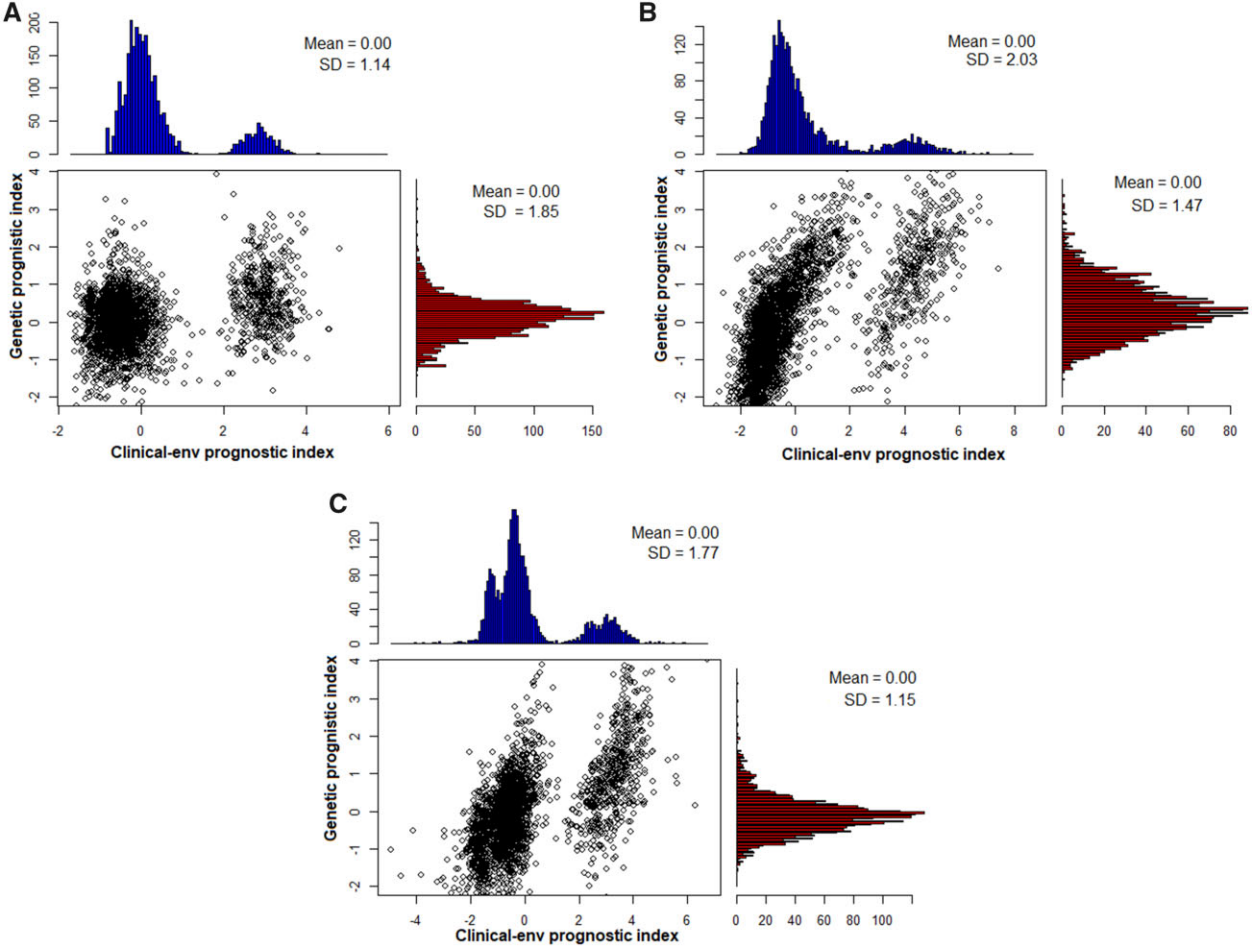
**Distributions of the prognostic index.** Panel (**A**) WoD, (**B**) RRE and (**C**) RWoD.

The scatter plots and histograms, alongside means and standard errors for the distributions of CEPI and GPI for each endpoint are shown on Fig. 1. They were constructed using the core clinical–env predictors (Table 1) and genetic variants (Supplementary Table 3). Whereas the GPIs are normally distributed, the CEPIs are mixtures of normal distributions that capture the complex heterogeneity of the MS disease course.

The correlations between the CEPIs and GPIs in each endpoint were estimated as follows: $\rho_{\mathrm{WoD}}$ = 0.61, $\rho_{\mathrm{RRE}}$ = 0.75 and $\rho_{\mathrm{RWoD}}$ =0.73, indicating a moderately high level of correlations between the clinical–env and genetic predictors at each survival endpoint. Meanwhile, a Cox regression on both the CEPI and GPI as predictors in each endpoint produced the CEGPIs as follows:

CEGPI=0.86*CEPI+0.56*GPI, if risk for WoD 0.80*CEPI+0.26*GPI, if risk for RRE 0.86*CEPI+0.27*GPI, if risk for RWoD.

Their respective standard deviations were estimated as $SD_{\mathrm{WoD}}\;$= 1.46, $SD_{\mathrm{RRE}}$ = 1.89 and $SD_{\mathrm{RWoD}}$ = 1.76.

### The predictive values of the prognostic indices

The log-hazard ratios (β), model-$\chi^{2}$, *pseudo-*$R^{2}$ and Harrell’s C-index ($\hat{C}$) for time-fixed Cox supermodels are given in Table 2. They confirm, respectively, the highest effect sizes, performances, variations and discrimination of the ‘super learners’ at each endpoint. The model parameters $\psi_{1}(\psi_{2})$ (Table 2) are, respectively, the calibrated clinical–env (genetic) effects in the CEGPI. The observation based on the model-$\chi^{2}$ (Table 2) when predicting the risk for WoD is that ≈27% [389/(389+1043)] of the overall prognostic information in the CEGPI is contributed by the genetic variants. For the RRE and RWoD endpoints, we observed ≃35% and ≃28% genetic contributions, respectively, further establishing the prognostic value of genetic variants for disease time-course predictions.

**Table 2:** Time-fixed supermodel Cox regression on cross-validation based clinical–environmental (CEPI), genetic (GPI) and clinical–environmental–genotype (CEGPI) prognostic indices. The *P*-values for all parameters were significantly less than $2\times 10^{-12}$

		**Worsening of disease (WoD)** N=2858;D=1011			**Relapses (RRE)** N=2858;D=564			**Relapses and/or worsening of disease (RWoD)** N=2858;D=1377		
Prognostic indices included	Parms. (Model)	β **(SE)**	($\hat{C}$, $R^{2}$)	**Model** $\chi^{2}$	β **(SE)**	($\hat{C}$, $R^{2}$)	**Model** $\chi^{2}$	β **(SE)**	($\hat{C}$, $R^{2}$)	**Model** $\chi^{2}$
Clinical–Env. (CEPI)	$\alpha_{1}\;(M_{4}$ )	0.96 (0.03)	(0.73, 0.57)	859	0.93 (0.03)	(0.85, 0.90)	1311	0.93 (0.05)	(0.76, 0.71)	1709
Genetic (GPI)	$\alpha_{2}\;(M_{5}$ )	0.86 (0.04)	(0.65, 0.32)	389	0.82 (0.05)	(0.79, 0.73)	746	0.84 (0.04)	(0.69,0.39)	680
	$\alpha_{1}\;(M_{6}$ )	0.86 (0.04)			0.80 (0.05)			0.86 (0.02)		
Both (CEPI and GPI)	$\alpha_{2}$	0.56 (0.07)	(0.76, 0.64)	1043	0.26 (0.06)	(0.85, 0.91)	1358	0.27 (0.06)	(0.77, 0.72)	1776
Clinical–Env–genotype (CEGPI)	$\alpha_{3}\;(M_{7}$ )	1.00 (0.03)	(0.76, 0.64)	1043	1.00 (0.03)	(0.85, 0.91)	1358	1.00 (0.03)	(0.77 0.72)	1776
Calibrated clinical	$\psi_{1}$	0.86/0.96 ≈**0.90**			0.80/0.93 ≈**0.86**			0.86/0.93 ≈**0.92**		
Calibrated genetic	$\psi_{2}$	0.56/0.86 ≈**0.65**			0.26/0.82 ≈**0.32**			0.31/0.85 ≈**0.36**		

$\psi_{1}=\;\mathrm{calibration}\;\mathrm{coefficient}\;\mathrm{for}\;\mathrm{CEPI};\;\beta =\;\mathrm{the}\;\mathrm{regression}\;\mathrm{coefficient};\;D=\;\mathrm{number}\;\mathrm{of}\;\mathrm{events};\;\;N=$ number of observations.

$\psi_{2}=\;\mathrm{calibration}\;\mathrm{coefficient}\;\mathrm{for}\;\mathrm{GPI};\;\hat{C}\;=$ Cross-validated Harrell’s C-index.

$\chi^{2}=\;\mathrm{model}\;\mathrm{chi}-\mathrm{square}\;\mathrm{statistics};\;R^{2}=\;\mathrm{pseudo}-R-\mathrm{square}\;\mathrm{computed}\;\mathrm{as}\;1-\exp({-\chi }^{2}/D)$ .

NB: In the column denoted ‘Parms’, the actual parameters in the supermodels are given. The results on this table were obtained from the fit of models $M_{4},\;M_{5},\;M_{6}\;\mathrm{and}\;M_{7,}$ respectively (see Supplementary methods).

CEPI: Clinical–Env Prognostic Index (clinical + environmental predictors).

GPI: Genetic Prognostic Index (Cumulative effects of single nucleotide polymorphisms markers).

CEGPI: Clinical–Env–Genotypic Prognostic Index = CEPI+GPI (clinical + environmental + genetic).

Time-fixed Supermodels ($M_{4},\;M_{5}\;\&\;M_{6}$): Cox regression performed on CEPI only, or GPI only, or combination of CEPI+GPI, without time-varying effects.

Super learner ($M_{7})\;\;\mathrm{Cox}\;\mathrm{regression}\;\mathrm{performed}\;\mathrm{on}\;\mathrm{CEGPI}\;\mathrm{only},\;\mathrm{after}\;\mathrm{obtaining}\;\mathrm{estimates}\;\mathrm{from}\;\mathrm{CEPI}\;(\alpha_{1})\;\mathrm{and}\;\mathrm{GPI}\;(\alpha_{2})\;\mathrm{from}\;\mathrm{model}\;M_{6}$.

**Figure 2:**
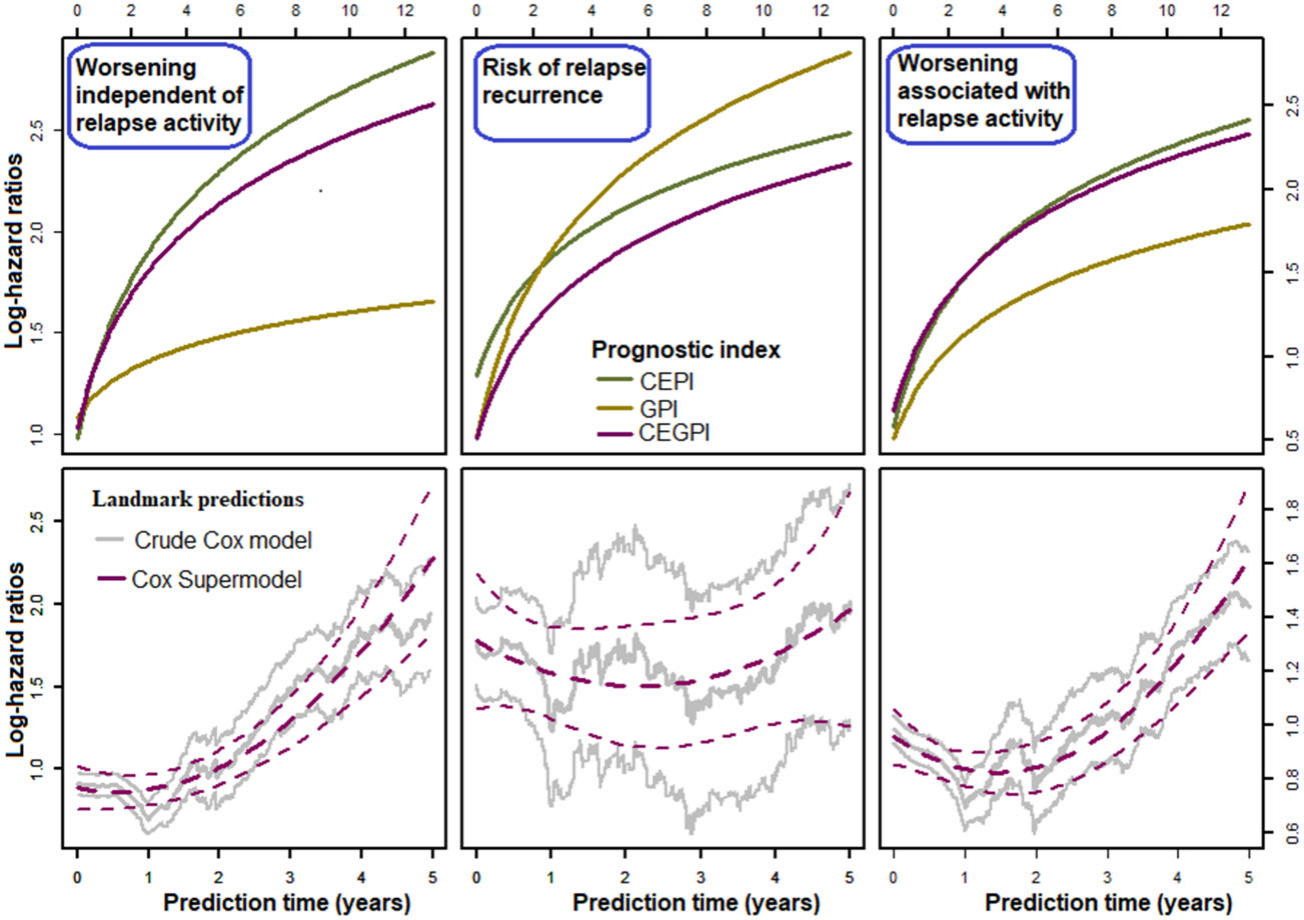
**Time-dependent regression effects of the prognostic index.** From left to right is the risk for WoD, RRE and RWoD, respectively.

**Figure 3:**
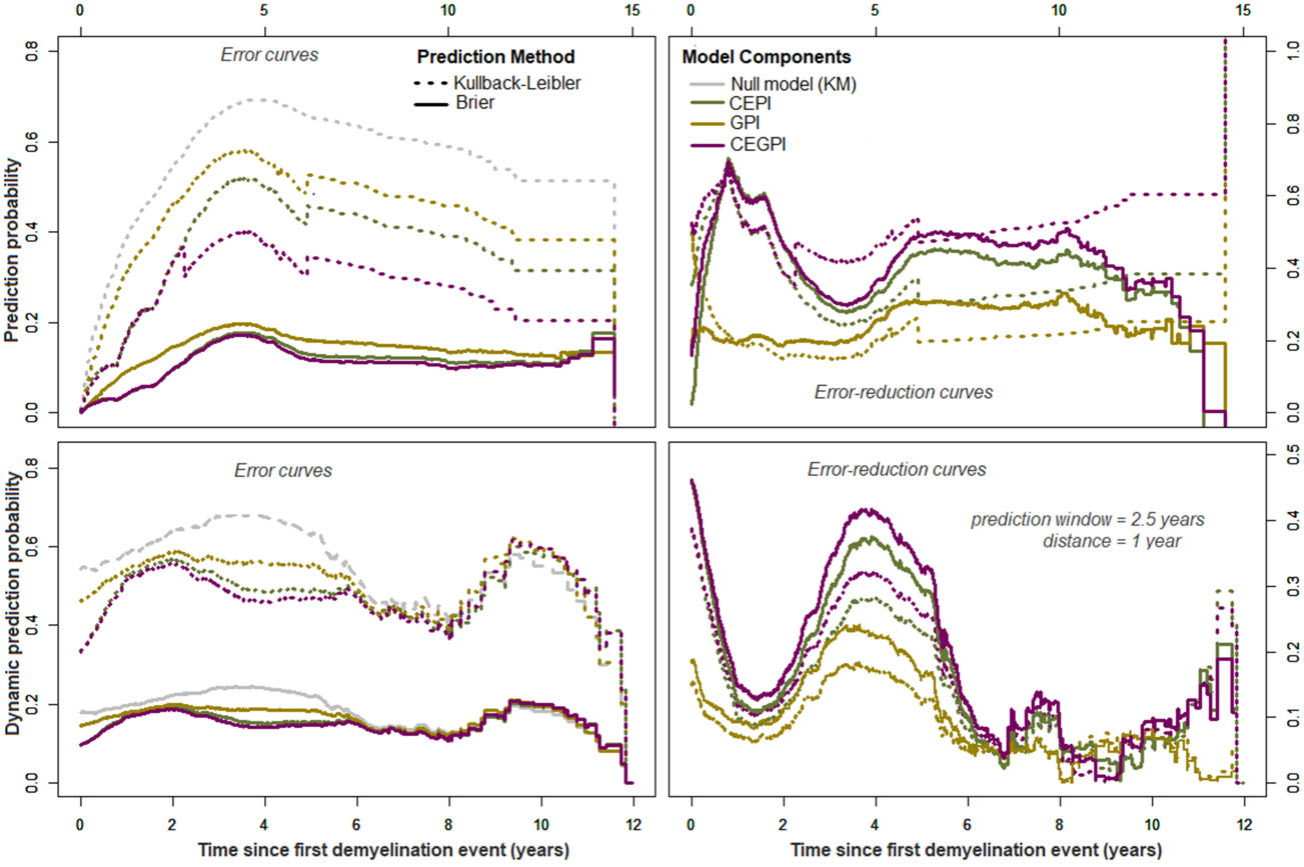
**Performance of the supermodels in predicting RWoD at diagnosis.** Prognostic errors and error-reduction probabilities based on the utility of the prognostic index.

**Figure 4:**
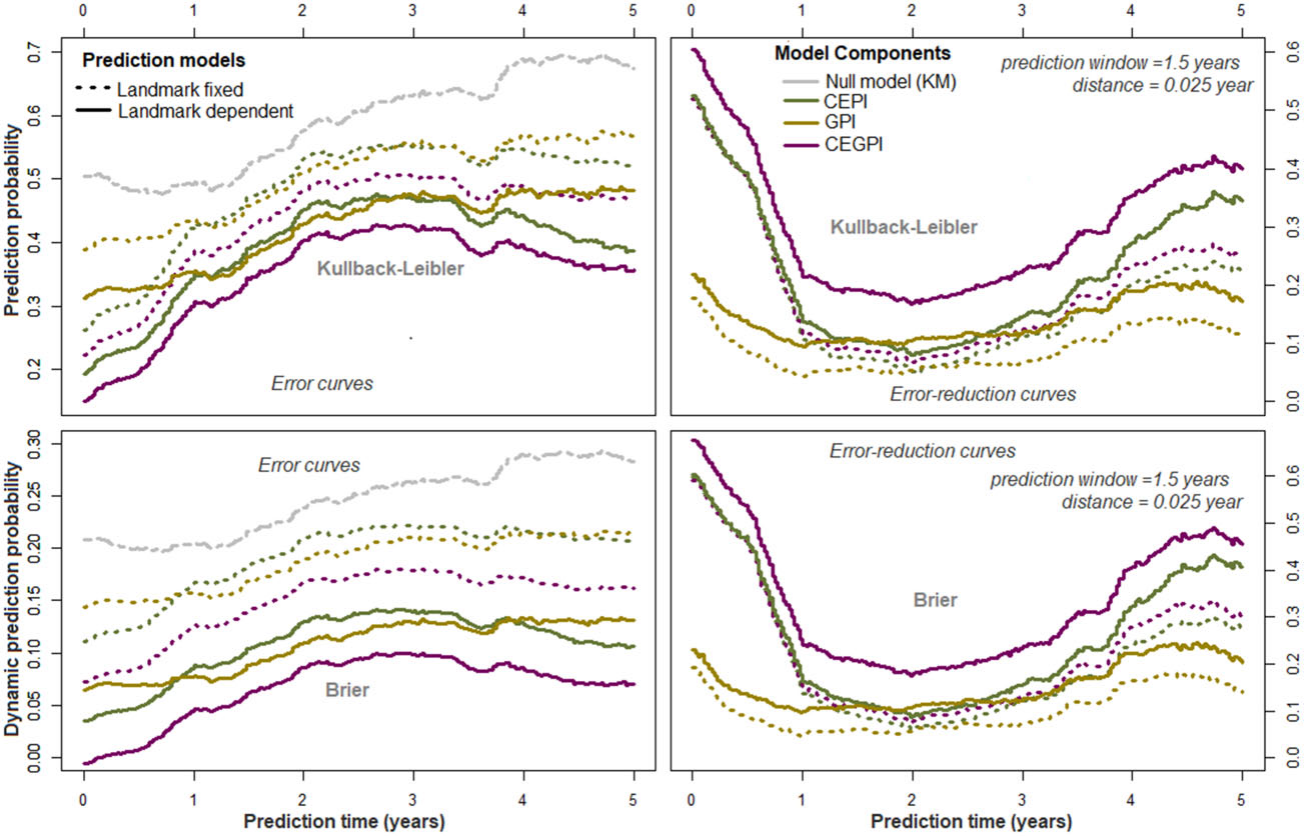
**Prospective accuracies of Landmark supermodels.** Prospective accuracies of Landmark supermodels in predicting RWoD within the next 5 years from diagnosis.

Meanwhile in the column denoted ‘time-varying’ (Table 3), the estimated time-dependent effects (ln(t+0.5)) of the prognostic indices are presented, and depicted graphically on Fig. 2 (top panels). Importantly, adjusting these time-varying effects through time-dependent Cox supermodels (‘time-varying’, Table 3), or Cox landmark supermodels (Supplementary Table 4) improved their predictive performance in terms of model-$\chi^{2}$ and prediction error probabilities (Figs 3 and 4). Regarding the predictive accuracies of the supermodels at diagnosis, the Kullback–Leibler and Brier (dynamic) prediction error (reduction) curves (Figs 3 and 4) suggest that for short-term prognosis (≤5 years), the clinical–env information is more relevant whereas the genetic information reduces the prediction error in the long-term (≥5 years). The ‘super learners’ perform better than the individual supermodels but not greatly so.

**Table 3:** Time-fixed and time-varying supermodel Cox regression on the clinical–environmental (CEPI), genetic (GPI) and clinical–env–genotype (CEGPI) prognostic index, respectively. Shown are the standard errors (SE) and the regression coefficients for the time-fixed effects (β ) and time-varying effects *ln(1+t)*. Non-significant effects have been highlighted

Prognostic index (PI)	**Model information**		**Constant** $\beta \left(\mathrm{SE}\right)$	**ln(t + 0.5)** β (SE)	**Model** $\chi^{2}$	Model AIC
Worsening of disease (N=2858; D=1011 )						
Clinical–Env. (CEPI)	$M_{4}$	Time-fixed	0.96 (0.03)		859	13757
		Time-varying	0.87 (0.06)	0.17 (0.09)	862	13756
Genetic (GPI)	$M_{5}$	Time-fixed	0.86 (0.04)		389	14227
		Time-varying	0.53 (0.09)	0.79 (0.17)	413	14204
Clinical–env–genotypic (CEGPI) $M_{6}=\;(0.86*\;\mathrm{CPI}+\;0.56*\;\mathrm{GPI}$)	$M_{7}$	Time-fixed	1.00 (0.03)		1043	13572
		Time-varying	0.92 (0.04)	0.16 (0.07)	1050	13568
Relapses (N=2858; D=564 )						
Clinical–Env. (CEPI)	$M_{4}$	Time-fixed	0.93 (0.03)		1311	7080
		Time-varying	0.88 (0.03)	0.17 (0.07)	1317	7076
Genetic (GPI)	$M_{5}$	Time-fixed	0.82 (0.05)		746	7645
		Time-Varying	0.76 (0.04)	0.28 (0.11)	727	7637
Clinical–env–genotypic (CEGPI) $M_{6}=\;(0.80*\;\mathrm{CPI}\;+\;0.26*\;\mathrm{GPI}$)	$M_{7}$	Time-fixed	1.00 (0.03)		1358	7033
		Time-varying	0.93 (0.03)	0.25 (0.07)	1369	7023
Relapses and/or worsening of disease (N=2858; D=1377 )						
Clinical–Env. (CEPI)	$M_{4}$	Time-fixed	0.93 (0.05)		1709	18162
		Time-varying	0.97 (0.04)	**−0.13 (0.08)**	1712	18162
Genetic (GPI)	$M_{5}$	Time-fixed	0.84 (0.04)		680	19192
		Time-Varying	0.77 (0.05)	0.24 (0.12)	684	19189
Clinical–env–genotypic (CEGPI) $M_{6}=\;(0.86*\;\mathrm{CEPI}\;+\;0.27*\;\mathrm{GPI}$)	$M_{7}$	Time-fixed	1.00 (0.03)		1776	18095
		Time-varying	1.02 (0.04)	**−0.06 (0.07)**	1777	18097

Supermodels ($M_{4},\;M_{5}\;\&\;M_{6}$): Cox regression performed on CEPI only, or GPI only, or CEPI+GPI.

Super learner ($M_{7}):\;\mathrm{Cox}\;\mathrm{regression}\;\mathrm{performed}\;\mathrm{on}\;\mathrm{CGPI}\;\mathrm{only},\;\mathrm{after}\;\mathrm{obtaining}\;\mathrm{estimates}\;\mathrm{from}\;\mathrm{CEPI}\;(\alpha_{1})\;\mathrm{and}\;\mathrm{GPI}\;(\alpha_{2})\;\mathrm{from}\;M_{6}$.

D= number of events, N= number of observations.

NB: The ‘time-fixed’/‘time-varying’ estimates were obtained from a Cox supermodel without/with time-varying effects, respectively. The ‘time-fixed’ estimates are identical to those found in Table 3 above. Adding the time-varying effects improved the performance of the time-vary supermodels over the time-fixed counterparts in terms of model chi-square ($\chi^{2}$) and AIC.

### Dynamic landmark predictions of disease course

The log-hazard ratios of the prognostic indices obtained from Cox supermodels performed on landmark datasets are shown in Supplementary Table 4. These results suggest that the effects of the clinical–env and genetic components increases with time. In terms of model-$\chi^{2}$ (Supplementary Table 4) and Kullback–Leibler information and Brier scores (Figs 3 and 4), these results further confirm the observation that the CEPIs performs slightly better than the GPIs, and the fact that the CEGPIs are not usefully better than the CEPI alone. In general, we obtained better discriminative capabilities with landmark-dependent, compared to landmark-fixed models (Fig. 4).

The robust estimates from the proportional baselines and stratified landmark supermodels are presented on Supplementary Table 5. Here, the effects of the CEPIs, GPIs, and hence the CEGPIs is the average over five landmark time points (i.e. average of Supplementary Table 4). In the column denoted ‘LM-fixed’ (Supplementary Table 5) the results of the landmark supermodels without interactions with landmark time points are reported, while in the columns denoted ‘LM-dependent’, the results are shown with linear (*s/5*) and quadratic (*s/5*)^2^ interactions with the landmark times, respectively.

**Figure 5:**
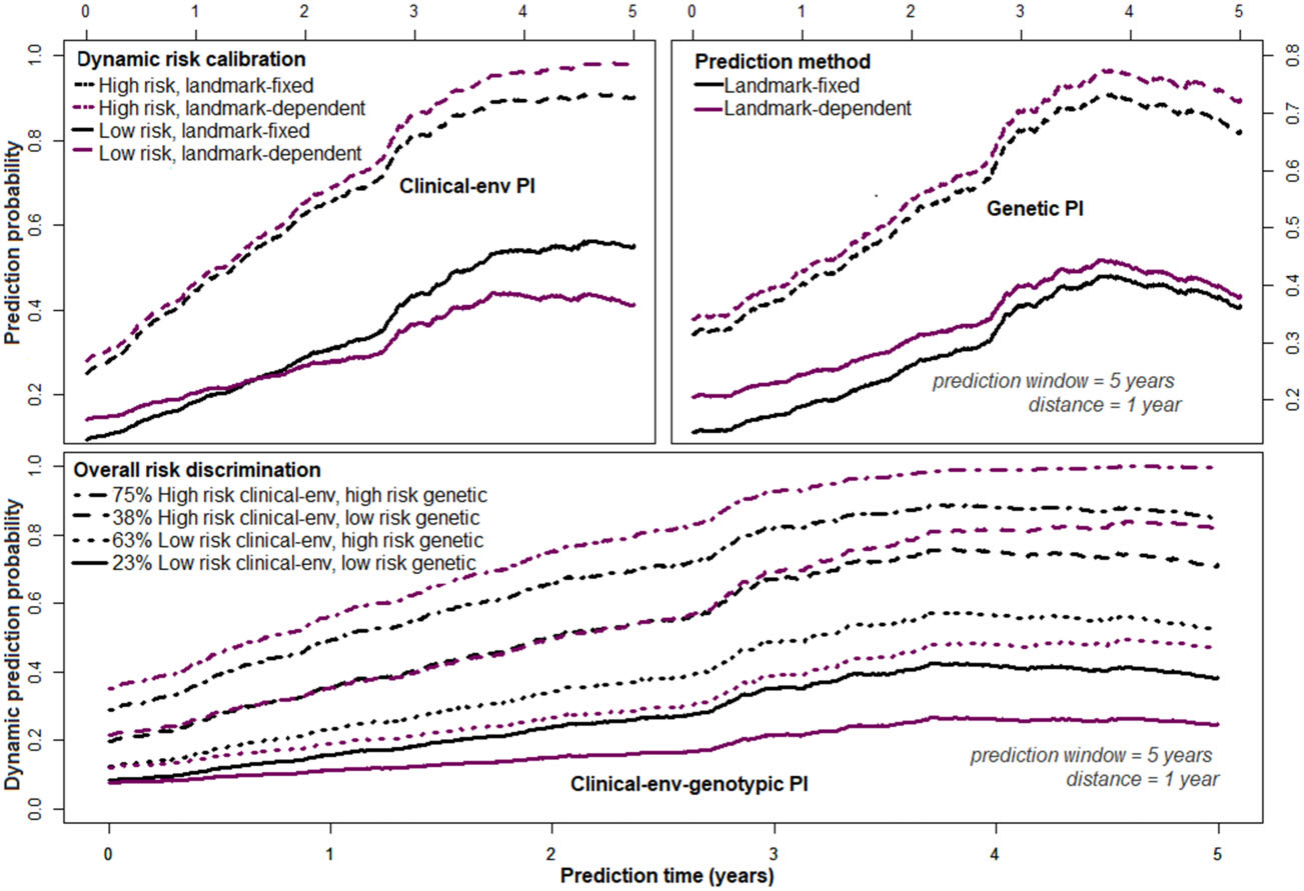
**Dynamic probability of having an RWoD event within the next 5 years.** Shown based on the Landmark approach.

The likelihood for risk of WoD, RRE and RWoD with regards to the quadratic of the CEGIPs effects (Fig. 2, bottom panels) is postulated to increase annually by 73% (HR = 2.74, 95% CI: 2.00–3.76), 68% (HR = 2.16, 95% CI: 1.74–2.68) and 67% (HR = 2.10 95% CI: 1.66–2.65), for 1unit change in the CEGPI, respectively. The 5 years dynamic prediction curves revealed four prognostic groups of MS cases (Fig. 5).

### Risk calibration using the prognostic index

**Figure 6:**
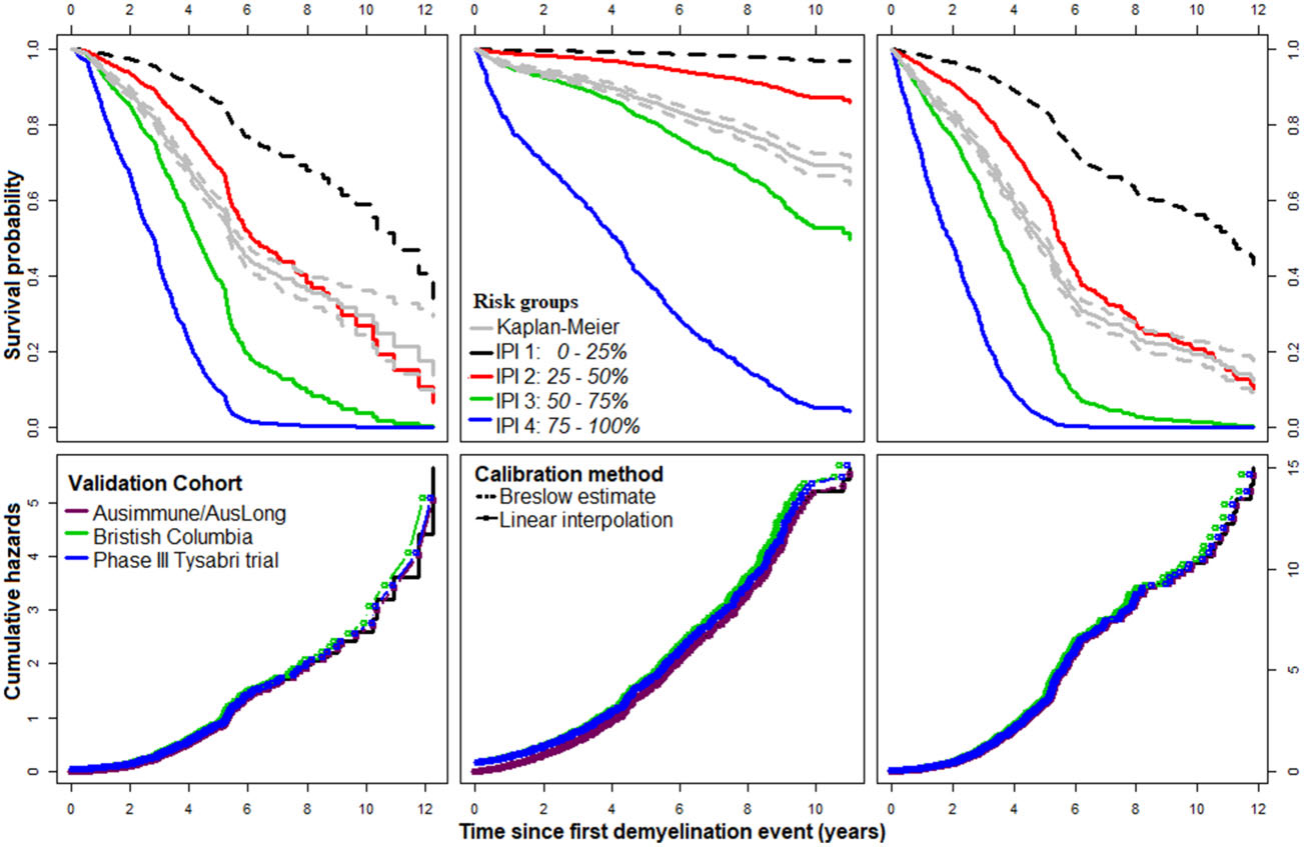
**Cross-validated Kaplan–Meier survival estimates based on the effects of the CEGPI.** From left to right: WoD, RRE and RWoD, respectively.

The estimated 5 and 10 years survival probabilities for the prognostic subgroups are shown on Supplementary Table 6. The hazards associated with the prognostic subgroups given the effects of the CEGPI are:

(3) $$\mathrm{WoD}:\;h\left(t|{\mathrm{IPI}}_{\mathrm{CEGPI}}\right)=h_{0}\left(t\right)\times \exp(1.50*\mathrm{IP}I_{2}+\;1.86*\mathrm{IP}I_{3}+3.08*\mathrm{IP}I_{4}$$

(4) $$\mathrm{RRE}:\;h\left(t|{\mathrm{IPI}}_{\mathrm{CEGPI}}\right)=h_{0}\left(t\right)\times \exp(0.96*\mathrm{IP}I_{2}+\;2.48*\mathrm{IP}I_{3}+4.03*\mathrm{IP}I_{4})$$

(5) $$\mathrm{RWoD}:\;h\left(t|{\mathrm{IPI}}_{\mathrm{CEGPI}}\right)=h_{0}\left(t\right)\times \exp(1.30*\mathrm{IP}I_{2}+\;1.46*\mathrm{IP}I_{3}+3.29*\mathrm{IP}I_{4})$$

where the low-risk group ($\mathrm{IP}I_{1}$) is the reference. The corresponding Kaplan–Meier curves for these subgroups are shown on Fig. 6 (top-panels). From these plots, it is clear that the CEGPIs are well calibrated in this data. In terms of baseline hazards, the CEGPIs are also well represented in the British Columbia cohort^53^ and the Phase III Tysabri trial^54^ (Fig. 6, bottom panels), although most likely applicable to the latter than the former, respectively.

## Discussion

In this study, we have developed 3 prognostic indices (CEPI, GPI and CEGPI) that can be applied to people with ROMS and CIS from diagnosis to 10 years of disease duration. We provided robust dynamic estimates for the risk of worsening, relapse and a combination of these metrics, respectively. The CEPIs provided the best discrimination between good and worse prognoses in the first 5 years of clinical symptoms, meanwhile the GPI had a greater effect after 5 years of symptomatic disease. The overall prognostic sensitivity was improved when using the combined index (CEGPI). The significant time-dependent effects of the prognostic indices enhanced their sensitivity for disease time-course predictions. These time-dependent effects strongly indicate that there are important variations in the drivers of MS progression, and therefore disease duration is an important variable in modelling MS progression. Importantly worsening events predicted the onset and recurrence of relapses, but the reverse was not supported. Interestingly, the genetic variants found to be significant in this study also interacted with latitude to increase the risk for worsening symptoms at higher latitudes.

Several clinical and environmental factors had significant time-varying effects on MS progression. Baseline T2L counts on MRI were a significant predictor of disease progression as previously described.^47^^,^^48^^,^^55^ Baseline BMI was borderline predictive of relapses, but had much stronger effects on worsening events. Regardless of previous EDSS and CDMS status, each $1\;\mathrm{kg}/m^{2}$ increase in BMI was associated with an 81% increased risk of worsening each year. Moreover, we noted that this effect was persistent up to 10 years post-FDE ($0.81^{10}=11\%$ risk), thus rendering it a good clinical marker for long-term prognostication. BMI, along with older age, income levels, smoking status and higher depression scores, has been shown to be associated with higher global disability in MS.^45^ Additionally, those taking vitamin D supplements had a better prognosis in terms of relapses or worsening of disability, but the effects of baseline 25(OH)D levels were marginal, and diminished significantly after 1 year post-onset. It is important to note that vitamin D supplementation at the time of this study largely consisted of VitD in multivitamin preparations and was in the range of 200–400 IU daily of vitamin D3.

Whether relapses are associated with worsening of disability has been an area of interest and some controversy in MS. In this cohort, individuals who presented with a ‘*worsening*’ clinical status at any given time, were on average, 56% more likely to develop relapses within the next year compared to those not worsening; whereas it was the relapse counts and not the relapse status that predicted worsening of disability. The overall finding in this direction is that ‘*worsening*’ events have stronger effects on relapse risk, but the reverse is less well supported. These observations are supported by previous studies.^53^^,^^56–58^ Importantly, the longer the duration between relapses (≥1 year between relapses), the greater is the reduction in the risk of future relapses and/or worsening symptoms.

We identified a limited number of genetic variations that predicted MS progression amongst those published by the International MS genetic consortium^43^ when constructing the GPIs. Particularly, SNPs that increased worsening risk such as *rs3819292*, *rs10951154*, *rs61863928*, *rs1112718* and *rs3184504*, had strong additive effects that tend to be protective for every $1^{0}$-degree increase in latitude. Conversely, those that had strong protective main effects such as *rs1177228*, *rs9878602*, *rs3923387*, *rs4409785* and *rs9955954*, interacted with latitude to increase worsening of disability each year. Therefore, these SNPs were associated with worsening symptoms mainly at higher latitudinal levels. These latitude-related genetic contributions are novel, and perhaps explain some of the genetic basis of high MS risk at higher latitudes found in this cohort.^59^^,^^60^

The clinical–env inputs were major contributors of disease progression, meanwhile the genetic inputs (although they had additional prognostic values) were minor contributors. These observations corroborate the views of Taylor.^23^ For instance, combining the effects of clinical–env predictors, and the genetic variations into a prognostic index improved the overall prediction accuracy as shown on Figs 3 and 4. The CEPIs alone explained ≃57%, 90% and 76% of the phenotypic variance in terms of WoD, RRE and RWoD, respectively. Given the CEPIs, the probability of correctly assigning higher risk scores to individuals with shorter times to events (Harrell’s C-index) were estimated as ${\hat{C}}_{\mathrm{WoD}}=73\%$, ${\hat{C}}_{\mathrm{RRE}}=85\%$ and ${\hat{C}}_{\mathrm{RWoD}}=76\%$. In contrast, a total of 25 SNPs (6-HLA and 19-non MHC autosomal) included in the GPI explained about 32% of the pure phenotypic variance in terms of worsening, with about 65% concordance among individuals. In terms of relapses, we included 61 SNPs (11-HLA and 50-non MHC autosomal) in the GPI to explain 73% of the phenotypic variance with about 79% concordance. Thus after adjusting for the effect of relapses (RWoD), the CEPI and GPI explained about 76% and 69% of phenotypic variance in WoD, respectively. Overall, prognostic predictions using the CEGPIs increased the phenotypic variations to 76% for WoD, 91% for RRE and 77% for RWoD. Additionally, we found ≤8% overlap between the genetic components of relapse and worsening, thus supporting our previous findings of independent genetic processes affecting relapse risk and disability worsening.^24^

Regarding the (dynamic) prediction errors, the CEPI alone were better for short-term prognostication (≤5 years from diagnosis) whereas the GPI reduced the errors only in the long-term (≥5 years from diagnosis). The CEGPI which combined the properties of the CEPI and GPI were suitable for both short- and long-term prognostications. However, its predictive accuracy depended on the time-varying effects of the clinical–env predictors included in the CEPIs. The underlying biological mechanism based on these findings is that the combined effects of the clinical–env predictors of disease were more variable at symptom onset compared to the effects of the genetic variations whose effects were pronounced only in the long-term. Over time, both components had differential interactions with disease duration to increase the risk of progression, thus explaining an overall quadratic time-dynamic disease course in terms of RWoD 5 years post-FDE as shown on Fig. 2 (bottom panels).

As pointed out in Henderson and Keiding,^61^ and stressed in Royston and Altman,^62^ prognostic models are not good at individual predictions for survival endpoints. Notwithstanding, we can still interpret the internal calibration curves of Fig. 6 (top panels) using prognostic models built on subgroups of individuals. The CEGPIs are capable of discriminating individuals having a poor prognosis (high-risk) from those having a good prognosis (low-risk). This is evident from the 5 and 10 years survival probabilities we presented on Supplementary Table 6. Importantly, individuals in the worst prognostic group (highest risk) had a 98%($\lambda_{HR}$=0.98) chance of having relapses within 1 year post-onset of symptoms. In this subgroup, we found, on average, 1.69 points (CI: 1.57–1.83) increment in EDSS per one-unit increase in the CEGPI value per annum, when compared to the baseline (low-risk). Similarly, we observed $\lambda_{\mathrm{HIR}}$=92% and $\lambda_{\mathrm{LIR}}$=72% chances of relapses within 1 year post-onset of symptoms in those having high- and low-intermediate risk, respectively. The hazards in these groups were obtained from prognostic model (4), and the risk scores λ were computed using equation (2).

The CEGPI could be used as a tool to stratify MS cases in future clinical trials, if its accuracy can be confirmed externally. Particularly to understand differential responses to therapies which may be influenced by the complex distributions of clinical–env factors, and genetic variations in this study cohort. If validated, it may be used as a tool for prognostication at an individual level to identify individuals who need greater surveillance and earlier use of more intensive therapy, and likewise in risk averse individuals. It may also provide some support for lesser interventions in those with a low-risk score.^21^^,^^63^^,^^64^ In this cohort, we have successfully identified and discriminated four groups of individuals based on their level of clinical–env and genetic risk they carry. These results are shown on Fig. 5. These scores have clinical implications, e.g. in treatment assignment (randomization) in clinical trial settings.

### Strengths and limitations

The most important limitation of this study is the lack of an external validation data set. It should be noted that the external *validation by calibration* curves on Fig. 4 (bottom panels) were linear interpolations of the baseline hazards. Since the AusLong study is an internationally unique first demyelination cohort, no external data were involved in the validation procedure. Another important limit is the sample size, and the restriction of the effect of duration of DMTs effect to baseline. It should be noted that DMTs were utilized at the discretion and timing of the clinician managing each MS case. Thus apart from the baseline measurements, the timings of the remaining follow-up were discrete and did not correspond to the true visits. As observed in each endpoint wherein the effects of the duration of DMTs trends towards the null hypothesis, had we used the complete follow-up measurements to account for its time-dynamic effects, we would expect strong beneficial effects in terms of relapses, and perhaps worsening of disability. This complex dynamic effect is clearly beyond the scope of this study and will be explored further. We also note that our CEGPI requires significant data including SNP data to be available for the index to be calculated which may limit its applicability in routine clinical practice, but should not be a concern in clinical trials as a stratification tool.

It should be noted that interactions at the level of the prognostic indices (CEPI × GPI) were not achievable due to the difficulties involved in the calibration of pure clinical–env effects ($\psi_{1}$ in Table 2) and pure genetic effects ($\psi_{2}$ in Table 2) in the combined index (CEGPI). Apparently, the information that is shared between the pure clinical–env source and the pure genetic one, and that is responsible for the correlation of the prognostic indices is much more relevant than the pure independent parts. However, had we included the interaction effect at the level of the prognostic index, then it will be practically impossible to disentangle the joint and independent effects of CEPI and GPI, separately. Thus, the decision to allow GxE interactions at the level of individual SNPs (SNP × Latitude) in the genetic models enabled us to properly calibrate and partial out the pure genetic effects (independent part) in each outcome while leveraging the latitude-related environmental contributions thereof. As such, a comprehensive assessment of GxE interaction at the level of the prognostic index is required. Last but not least, our findings that latitude interacts with the genetic variants indicate this is a fruitful area for further research.

### Declaration of good modelling practice

We used statistical analysis methods published in internationally reputable statistical journals ^25^^,^^26^^,^^28^^,^^36^^,^^39–42^^,^^51^^,^^52^ to analyse disease progression in this cohort. Outcome measures such as the time to 3 or 6 months confirmed disability progression, or annualized change in EDSS have been strongly criticized for assuming the underlying MS disease course evolves in discrete-time intervals, meanwhile the real biological process of MS disease (EDSS transitions) evolves in continuous-time^35^^,^^36^^,^^65^^,^^66^; and for overestimating permanent disability when used in short-term clinical trials.^67^ Following this, our definition of WoD in this study was based on restructuring of the data following Markov assumptions.^35^^,^^36^ In this way, the ordinal nature of EDSS was preserved, and we analysed the probability, at any given time, of observing the current EDSS given the entire history. A continuous duration of the disease rather than discrete-time (actual visits) was considered when restructuring the data and modelling of progression process.

## Conclusion

In this cohort, we have created a CEGPI for individuals with ROMS and CIS, taking into account the multifactorial nature of MS disease course. We obtained robust dynamic predictions for the probability of developing new relapses and worsening of disability. The CEGPIs ability to reliably discriminate individuals with a higher risk of worsening potentially makes it a useful prognostic tool for estimating a person’s probability of developing a worse MS course at diagnosis. Although the genetic variations provided additional prognostic values for disease time-course prediction, the clinical and environmental components were the major contributors. Our CEGPI provided reliable information that is relevant for long-term prognostication, but is more applicable as a clinical research tool. If externally validated, it may be used in risk stratification and selection criteria in clinical trials.

## Supplementary material

Supplementary material is available at *Brain Communications* online.

## Supplementary Material

fcab288_Supplementary_Data

## Appendix I

**Ausimmune/AusLong Investigators Group members:** RL (National Centre for Epidemiology and Population Health, Canberra), Keith Dear (Duke Kunshan University, Kunshan, China), A-LP and Terry Dwyer (Murdoch Childrens Research Institute, Melbourne, Australia), IvdM, LB, SSY, BVT, and Ingrid van der Mei (Menzies Institute for Medical Research, University of Tasmania, Hobart, Australia), SB (School of Medicine, Griffith University, Gold Coast Campus, Australia), Trevor Kilpatrick (Centre for Neurosciences, Department of Anatomy and Neuroscience, University of Melbourne, Melbourne, Australia). David Williams and Jeanette Lechner-Scott (University of Newcastle, Newcastle, Australia), Cameron Shaw and Caron Chapman (Barwon Health, Geelong, Australia), Alan Coulthard (University of Queensland, Brisbane, Australia), Michael P Pender (The University of Queensland, Brisbane, Australia) and Patricia Valery (QIMR Berghofer Medical Research Institute, Brisbane, Australia).

## Abbreviations

AusLong =: the Ausimmune/Auslong study
BMI =: body mass index
CDMS =: clinically defined multiple sclerosis
CEPI =: clinical–environmnetal prognostic index
CEGPI =: clinical–environmental–genotypic prognostic index
CIS =: clinially isolated syndrome
CIs =: confidence intervals
DMTs =: disease modifying therapies
EDSS =: expanded disability status scale scores
FDE =: first demyelination event
GPI =: genetic prognostic index
HADS =: hospital anxiety depression scores
HLA =: human leukocyte antigen
LASSO =: least absolute shrinkage and a selection operator
LOOCV =: leave-one out cross-validation
MHC =: major histocompatibility complex
MS =: multiple sclerosis
ROMS =: relapsing-onset multiple sclerosis
RRE =: recurrent relapsing events
RWoD =: relapse and/or worsening of disability
SNPs =: single nucleotide polymorphisms
T2L =: T2 brain MRI lesion count (T2 lesions for short)
WoD =: worsening of disability
