# Supplementary material for: Developing a clinical–environmental–genotypic prognostic index for relapsing-onset multiple sclerosis and clinically isolated syndrome
Source: Brain Commun. 2021 Dec 4;3(4):fcab288. doi: 10.1093/braincomms/fcab288 (PMC8691056; doi:10.1093/braincomms/fcab288)
Supplement: fcab288_Supplementary_Data [file fcab288_supplementary_data.zip › Supplementary_Methods_VFN.docx]

**Supplementary Methodology to accompany the article:**

***“Developing a Clinical-Environmental-Genotypic Prognostic Index for Relapsing-Onset Multiple Sclerosis and* Clinically Isolated Syndrome”.**

**Main Author:** Valery Fuh-Ngwa

**Email**: [valeryfuh.ngwa@utas.edu.au](mailto:valeryfuh.ngwa@utas.edu.au)

**Address**: Menzies Institute for Medicval research,

17 Liverpool St, Hobart TAS 7000.

- 1. **Screening of informative SNPs.**

For each survival endpoint (RRE, WoD, RWoD), an initial global test for the predictive significance of all SNPs was done using the Goeman’s “$globaltest$” R-package,^1^ in which additive genetic models were assumed for all 199 SNPs. Next, a time-dependent penalized Cox-regression model with least absolute shrinkage (Cox-Lasso) was then employed to select a limited number of SNPs using the Goeman’s “$penalized$” R-package.^2^ In particular, for each survival endpoint, we posit that

$h_{j}\left( t | X_{j} \right)=h_{0}\left( t \right)\exp\left( \beta X_{j}^{T} \right)$ $(1.1)$

where $h_{j}(t)$ is the hazard function for the $j$-th subject; $h_{0}$ is a common baseline hazard function, and $\beta$ is a vector of fixed effects regression coefficients associated with the genotype matrix $X_{j}^{T}$. Penalizing the partial log-likelihood by a factor $\lambda*\sum|\beta|$ resulted in absolute shrinkage of the $\beta$ coefficients, and hence the selection of a limited number of SNPs for prognostic analysis ^3,4^. That is

$$pI_{lasso}\left( \beta\right)=\sum_{j=1}^{n} \delta_{j}*\log\left( \frac{\exp\left( \beta X_{j}^{T} \right)}{\sum_{i\in R\left( t_{j} \right)} \exp\left( \beta X_{j}^{T} \right)} \right) -\frac{1}{\lambda}\sum_{j=1}^{p} \left| \beta_{j} \right|, \lambda\geq0$$

where $pI_{lasso}(\beta)$ is the Cox-Lasso estimator for the partial log-likelihood of $\beta$, induced by the Breslow estimator log(.). Optimal values for penalty parameter $\lambda$ were then chosen by leave-one-out cross-validation.

Unbiased estimates for $pI_{lasso}(\beta)$ and their standard errors were obtained by re-estimating the effects of the Lasso-selected SNPs using the “mfp” R-package implementing the method of Sauerbrei and Royston.^5^ To adjust for differences in actual risk due to differences in EDSS and variability in the time to conversion (CDMS), EDSS and CDMS were included as stratification variables, and the robust option under the Anderson-Gill (AG) modeling framework^6^ was adopted to adjust for correlated observations. The results generated from the above procedure are presented on Supplementary Table 3.

## **Screening of clinical and environmental predictors.**

Initial screening of possible predictors for each endpoint was based on evidence from previous studies (Supplementary Table 1). To get an insight into the prognostic effects of each predictor process across the endpoints, a crude Cox model was fitted using all available predictors. This was achieved using “survival” R-package within AG framework.^6^ In particular, a model of the form

$h_{j}\left( t | Z_{j} \right)=h_{0}\left( t \right)\exp\left( \gamma Z_{j}^{T} \right)$ $(1.2)$

was fitted to the data. Here $\gamma$ is a vector of regression coefficients associated with a $q$-dimensional matrix of clinical predictors $Z_{j}^{T}$. The core models were then obtained according to Sauerbrei and Royston ^5^. Here a multifactorial search for fractional polynomials was allowed. The results of the core and crude Cox models have been retained on Main article Table 1 and Supplementary Table 2, respectively.

## **Synthesizing the clinical-env-genotypic prognostic index.**

To assess whether the genetic variants have an added predictive value to the clinical predictors, and which approach is best able to do so, the clinical predictor matrix $Z_{j}^{T}$ could be included directly into the Lasso selection model (1.1) via the Goeman’s “$penalized$” R-package, in which a model of the form

$h_{j}\left( t | X_{j}, Z_{j} \right)=h_{0}\left( t \right)\exp\left( X_{j}^{T}\beta(t)+Z_{j}^{T}\gamma(t) \right)$ $(1.3)$

is fitted to the data. However, a potential drawback of the model (1.3) is that the correlation between the genetic variants and clinical-env predictors can lead to dramatic changes in both $\beta$ and $\gamma$ compared with the models with only clinical-env or only genetic variants.^7^ To remedy this situation for each survival endpoint, we posit that

$M_{1}$ $h_{j}\left( t | \mathbf{Z}_{j} \right)=h_{0}\left( t \right)\exp\left( \mathbf{Z}_{j}^{T}\boldsymbol{\gamma(t)} \right)$ leading to $\mathbf{CEPI(Z}\boldsymbol{)}$

$M_{2}:$ $h_{j}\left( t | X_{j} \right)=h_{0}\left( t \right)\exp\left( \mathbf{X}_{j}^{T}\boldsymbol{\beta(t)} \right)$ leading to $\mathbf{GPI(X)}$

$M_{3}:$ $h_{j}\left( t | \mathrm{PI} \right) =h_{0}\left( t \right)\exp\left( \alpha_{1}\mathbf{CEPI}\left( \mathbf{Z} \right)+\alpha_{2}\mathbf{GPI}\left( \mathbf{X} \right) \right)$ leading to $\mathbf{CEGPI(X, Z)}$

where $\mathbf{CEPI(Z)}$ is the clinical-env prognostic index (PI) obtained from a matrix $Z_{j}^{T}$containing the clinical-env predictors; **GPI**$\mathbf{(X)}$ is the genetic PI obtained from a SNP matrix $X_{j}^{T}$, containing both the main and interaction effects. In particular, interaction with time and standardized latitudinal coordinates; and $\mathbf{CEGPI(X,Z)}$ is the clinical-env-genotypic PI derived from a linear combination of the $\mathbf{CEPI(Z)}$ and **GPI**$\mathbf{(X)}$. The combined index ($\mathbf{CEGPI(X,Z)}$) is very similar in the spirit of the combination of models and the “super learner “ of van der Laan et al.,^8^ and van Houwelingen and Putter.^7^ Henceforth, we refer to Cox models fitted with the PI as the main predictor as “supermodels”, and the models fitted with $\mathbf{CEGPI(X,Z)}$) alone as super learners.

### **Internal validation based on cross-validation.**

To avoid model overfitting, the parameters in $M_{3}$ were estimated using the cross-validated versions of the PIs. To achieved this, the cross-validation based predicted survival curves for the j-th subject were computed from the core genetic and clinical-env survival models $M_{1}\mathrm{and}M_{2}$ as

$\hat{S}_{gen,CV, j}\left( t \right)= \hat{S}_{\left( -j \right)}\left( t | \boldsymbol{X}_{j}^{T} \right)=exp[-\hat{H}_{0,\left( -j \right)}\left( t \right) exp(X_{J}^{T} \hat{\beta}_{\left( -j \right)})]$ $(2.1)$

$\hat{S}_{clin,CV, j}\left( t \right)= \hat{S}_{\left( -j \right)}\left( t | Z_{j}^{T} \right)=exp[-\hat{H}_{0,\left( -j \right)}\left( t \right) exp(Z_{J}^{T} \hat{\gamma}_{\left( -j \right)})]$ $(2.2)$

and the cross-validation based genetic and clinical PIs using

$GPI_{CV} =PI_{gen,j,\left( - j \right)}= \sum_{j=1}^{n} X_{j}^{T}\hat{\beta}_{\left( -j \right)}$ $(2.3)$

$CEPI_{CV} =PI_{clin,j,\left( -j \right)}= \sum_{j=1}^{n} Z_{j}^{T}\hat{\beta}_{(-j)}$ $(2.4)$

respectively. Notably, the predicted survival curves in (2.1) and (2.2) were invariant under additive transformations of the genetic *X* and the clinical-env *Z* predictor matrices, whereas their cross-validation based PIs were not.^7^ Based on this observation, we defined a slightly more elegant version for each PI as

$CEPI_{CV} =PI_{clin, CV, j}=\ln\left( -\ln\left( \hat{S}_{clin, CV, j}\left( t \right) \right) \right)$ $(2.5)$

$GPI_{CV} =PI_{gen, CV, j}=\ln\left( -\ln\left( \hat{S}_{gen,CV, j}\left( t \right) \right) \right)$ $(2.6)$

with

$CEGPI_{CV}=\alpha_{1}*CEPI_{CV}+$ $\alpha_{2}*GPI_{CV}$ $(2.7)$

which were not invariant under scaling of the genetic **X** and clinical **Z** matrices, as they depend intrinsically on $\boldsymbol{t}$. The centered versions of the cross-validated PIs in (2.5) to (2.7) were then used for model validation and model comparison. The cross-validation based genetic ($GPI_{CV}$) is then considered as the cumulative adverse genetic effects conferred by all the SNPs that were predictive of the disease status, and is estimated for each subject at event time point $\boldsymbol{t}$. A similar argument holds for the cross-validation based clinical ($CEPI_{CV}$), as the marginal contributions from all clinical features. Hence, the computation of PI at time $t$ implies a time-varying PI.

## **Dynamic predictions with time-varying prognostic indices.**

- - 1. **Dynamic predictions on overall dataset.**

To get a useful insight into the biological mechanisms, that is, which aspects of the genetic and clinical-env predictor processes drive the hazards, and whether the effects on the hazards varies with time, we postulate the following time-dependent Cox supermodels for

$M_{4}: h_{j}\left( t | PI(t) \right)=h_{0}\left( t \right)\exp\left( \mathrm{CEPI}\left( t \right)\alpha_{1} \right)$

$M_{5}:$ $h_{j}\left( t | PI(t) \right)=h_{0}\left( t \right)\exp\left( \mathrm{GPI}\left( t \right)\alpha_{2} \right)$

$M_{6}:$ $h_{j}\left( t | PI(t) \right)=h_{0}\left( t \right)\exp\left( \mathrm{CEPI}\left( t \right)\alpha_{1}+GPI\left( t \right)\alpha_{2} \right)$

$M_{7}$: $h_{j}\left( t | PI(t) \right)=h_{0}\left( t \right)\exp\left( {\mathrm{CEGPI}\left( t \right)\alpha}_{3} \right)$

each survival endpoint, where $\psi_{1}=\alpha_{1\left( M_{4} \right)}/\alpha_{1(M_{6})}$, and $\psi_{2}=\alpha_{2\left( M_{5} \right)}/\alpha_{1(M_{6})}$ are respectively, the calibrated effect of CEPI and GPI in the CEGPI. These quantities are given on Table 3. The clinical-env-genotypic supermodel $M_{6}$ (used to derived $\mathrm{CEGPI}$) gives an unbiased view on the contribution of the two sources of biological information and is captured by the super learner $M_{7}$. The results from the fit of models $M_{3}$, $M_{4}$ and $M_{7}$ has been retained on Table 3.

- - 1. **Dynamic prediction by Landmarking.**

Dynamic prediction by landmarking is well elaborated for instance in.^7-10^ Since the models stated from $M_{4}\mathrm{to}M_{7}$ could not be used for prognostic assessments, we opt to obtain sliding landmark robust dynamic predictions for this data. In particular, we created landmark data sets at equally-spaced medically relevant time points $\mathbf{t}_{\mathbf{LM}}\mathbf{=}$ 0, 1, 2, 3, 4, and 5 years, using a relevant prediction window of width $\boldsymbol{w}=5$ years, running 1 year distance apart. For a single landmark and each survival endpoint, we posit that

$M_{4}^{'}$: $h_{s}\left( t | \mathrm{PI}\left( s \right), \mathbf{w} \right) = h_{s,0}\left( t \right)\exp\left[ CEPI(s)\boldsymbol{*\alpha}_{1s} \right]$

$M_{5}^{'}$: $h_{s}\left( t | \mathrm{PI}\left( s \right), \mathbf{w} \right) = h_{s,0}\left( t \right)\exp\left[ GPI(s)\boldsymbol{*\alpha}_{2s} \right]$

$M_{6}^{'}:$ $h_{s}\left( t | PI(s), \mathbf{w} \right) =h_{s,0}\left( t \right)\exp\left( \mathrm{CEPI}\left( s \right)\boldsymbol{*}\alpha_{1s}+GPI\left( s \right){*\alpha}_{2s} \right)$

$M_{7}^{'}$: $h_{s}\left( t | \mathrm{PI}\left( s \right), \mathbf{w} \right) = h_{s,0}\left( t \right)\exp\left[ \mathrm{CEGPI}\left( s \right){*\alpha}_{3s} \right]$

$\mathrm{for}s\leq t\leq s+\mathbf{w}$**,**

where the regression coefficients $\alpha_{\left( . \right)}$ depends only on the information available at $s={[s}_{1}, \ldots, s_{L}]$, with $s_{L}=5 years$. Note that the $CEPI(s)$ and $GPI(s)$ in $M_{4}^{'} \& M_{5}^{'}$ are respectively the cross-validation based clinical-env and genetic PIs derived from different Landmark data sets. Their computations are straightforward and follow from Eqn. (2.5), that is

$CEPI(s)=\ln\left( -\ln\left( \hat{S}\left( s+t \right) | s, \boldsymbol{Z} \right) \right)$ $(2.8)$

$GPI(s)=\ln\left( -\ln\left( \hat{S}\left( s+t \right) | s, \boldsymbol{X} \right) \right)$ $(2.9)$

which are both well-defined for every Landmark time point $s$.^7^ Similarly, the clinical-env-genotypic PI ($\mathrm{CEGPI}(s)$) in $M_{7}^{'}$ is obtained from $M_{6}^{'}$ after a cox regression on cross-validated versions of CEPI(s) and GPI(s). For instance, at landmark s=0, the CEGPI(s=0) is computed as

$\mathrm{CEGPI}\left( s=0 \right)=[\mathrm{CEPI}(s=0)*\alpha_{1\left( s=0 \right)}$ $+ GPI(s=0)*\alpha_{2(s=0)}]$ (3.0)

For subsequent landmark time points $s\geq1$, we substituted s= (1, 2…, 5). The “time-fixed” estimates are presented on main article Table 2. The results from the fit of models $M_{4}^{'}$, $M_{5}^{'}$, and $M_{7}^{'}$ are given on main article Table 3. Note that $M_{6}^{'}$ was only use to obtain $M_{7}^{'}$, hence results for $M_{6}^{'}$ were not shown, but the estimated effects of $\alpha_{1}\& \alpha_{2}$ are have been retained on main text Table 3 (first column).

- 1. **Model Validation, Discrimination, and Predictive Performance**

## **Predictive Performance**

The predictive performance for each supermodel was assessed using the $X^{2}$-statistics and the dynamic area under the curve (AUC(t)). Next, a visual check of the model validity was done for each survival endpoint by simply comparing the predicted survival curves with Kaplan-Meiers (KM) in subgroups based on cross-validated PIs. This was achieved by stratifying subjects into four groups of equal sizes based on risk quantiles of. the so-called international PI, and then estimated the survival within each group by performing a traditional cox-regression with the subgroups as covariates. Following this, the overall performance for each model at diagnosis was based on visual checks using the moving window Kullback-Leibler and Brier dynamic prediction error (reduction) curves for both the separate and combined models respectively. These results have been captured in Fig. 3.

## **Internal Calibration by Shrinkage**

To generalized the predictive performance beyond forming prognostic subgroups, we adopted the calibration modeling approach of van Houwelingen and Putter H.^7^ in which the predictive performance of the PIs was improved by shrinking towards its mean. Specifically, we defined a heuristic shrinkage factor $0\leq\hat{c}\leq1$ as

$$\hat{c}=1-\frac{\dim}{X_{\mathrm{model}}^{2}}$$

where $\dim$ is the dimension of the prediction model, and $X_{\mathrm{model}}^{2}$ is the model chi-square statistics computed as ${2*(l}_{\mathrm{model}}-l_{0}$) (where $l_{0}$ is the likelihood of the null model). For each survival endpoint, optimal values for $\hat{c}$ were chosen by cross-validation. Truly-speaking, $\hat{c}$ measures the goodness-of-calibration, that is how well the models discriminate between subjects that had the event from those that did not. Similar to Harrell’s C-index, higher values of $\hat{\mathbf{c}}$ close to 1 results in well-calibrated models with reduced prediction errors.^7,11^

## **External Validation by Calibration.**

To check whether the PIs developed in our data were valid for an external population, we adopted the *validation by calibration* approach used in^12^ in which a calibration supermodel of the form

$\ln\left( H_{cal}\left( t | \mathrm{PI} \right) \right)=\theta_{0}+\theta_{1}\mathrm{PI}+\theta_{2}ln(H_{0}^{*}(t))$ $(3.1)$

was defined for $\mathrm{CPI}$, $\mathrm{GPI}$, and $\mathrm{CGPI}$, respectively. The corresponding cumulative hazard is given by $H^{*}\left( t | \mathrm{PI} \right)=\exp\left( \mathrm{PI} \right)\times H_{0}^{*}(t)$, with $H_{0}^{*}(t)$ as a baseline hazard from an external population. Since the baseline hazard from the external population is often not reported in publications on prediction models, we decided to fit a *Weibull calibration* model to test whether the shape of the baseline hazard in our data were correct. To achieve this, a model of the form

$\ln\left( H\left( \tilde{t} | PI \right) \right)=\theta_{0}+\theta_{1}\mathrm{PI}+\theta_{2}ln(\tilde{t})$ $(3.2)$

is fitted on our data, in which $\tilde{t}={\hat{\alpha} H}_{model, i}^{\beta}\left( t \right)$. Here $\hat{\alpha}=\sum\delta_{i}/\sum H_{0}^{*}(T_{i})$ is the standardized mortality ratio (SMR) calculated based on the parameters reported in the external population, $H_{model, i}(t)$ and $\beta$ are respectively the baseline hazard and regression coefficient of the PI estimated in our data.

By realizing that the standard error of SMR takes the form $se\left( \ln\left( \hat{\alpha} \right) \right)=exp(-\hat{\alpha})=1/\surd(\sum\delta_{i})$, the SMR can be obtained using $\hat{\alpha}=-\ln(se\left( \ln\left( \hat{\alpha} \right) \right))$. Information on the SMRs was obtained from two external populations namely, the British Columbia cohort reported in^13^, and the Phase III Tysabri trial of MS from North America.^14^ Logically-speaking, the SMR is considered as an estimate for the approximation of the baseline hazards in these populations for situations where the total event time $\sum H_{0}^{*}(T_{i})$ has not been reported. The effort of this calibration is shown in the main text Fig. 6 and Supplementary Fig 1.

**Supporting Literature**

1. Goeman JJ, Van De Geer SA, De Kort F, Van Houwelingen HC. A global test for groups of genes: testing association with a clinical outcome. *Bioinformatics.* 2004;20(1):93-99.

2. Goeman JJ, Oosting J, Cleton-Jansen A-M, Anninga JK, Van Houwelingen HC. Testing association of a pathway with survival using gene expression data. *Bioinformatics.* 2005;21(9):1950-1957.

3. Le Cessie S, Van Houwelingen JC. Ridge estimators in logistic regression. *Journal of the Royal Statistical Society: Series C (Applied Statistics).* 1992;41(1):191-201.

4. Van Houwelingen HC, Bruinsma T, Hart AAM, Van'T Veer LJ, Wessels LFA. Cross-validated Cox regression on microarray gene expression data. 2006;25(18):3201-3216.

5. Sauerbrei W, Royston P. Building multivariable prognostic and diagnostic models: transformation of the predictors by using fractional polynomials. 1999;162(1):71-94.

6. Andersen PK, Gill RD. Cox's Regression Model for Counting Processes: A Large Sample Study. *Ann Statist.* 1982;10(4):1100-1120.

7. van Houwelingen H, Putter H. *Dynamic prediction in clinical survival analysis.* CRC Press; 2011.

8. Van der Laan MJ, Polley EC, Hubbard AE. Super learner. *Stat Appl Genet Mol Biol.* 2007;6(1).

9. Van Houwelingen HC. Dynamic Prediction by Landmarking in Event History Analysis. *Scandinavian Journal of Statistics.* 2007;34(1):70-85.

10. Van Houwelingen HC, Putter H. Dynamic predicting by landmarking as an alternative for multi-state modeling: an application to acute lymphoid leukemia data. *Lifetime Data Analysis.* 2008;14(4):447-463.

11. Van Houwelingen J, Le Cessie S. Predictive value of statistical models. *Statistics in medicine.* 1990;9(11):1303-1325.

12. van Houwelingen HC. Validation, calibration, revision and combination of prognostic survival models. *Statistics in Medicine.* 2000;19(24):3401-3415.

13. Tremlett H, Yousefi M, Devonshire V, Rieckmann P, Zhao Y, Neurologists UBC. Impact of multiple sclerosis relapses on progression diminishes with time. *Neurology.* 2009;73(20):1616-1623.

14. Wang YC, Meyerson L, Tang YQ, Qian N. Statistical methods for the analysis of relapse data in MS clinical trials. *Journal of the Neurological Sciences.* 2009;285(1):206-211.
