# Supplementary material for: Developing a clinical–environmental–genotypic prognostic index for relapsing-onset multiple sclerosis and clinically isolated syndrome
Source: Brain Commun. 2021 Dec 4;3(4):fcab288. doi: 10.1093/braincomms/fcab288 (PMC8691056; doi:10.1093/braincomms/fcab288)
Supplement: fcab288_Supplementary_Data [file fcab288_supplementary_data.zip › Supplementary_Tables_VFN.docx]

**Supplementary Table 1.** Potential clinical, environmental, and demographic predictors of multiple sclerosis disease progression outcomes: Ausimmune/AusLong cohort characteristics for those who completed the 5^th^ and 10^th^ -year reviews. The sample size (n) and percentages (%) is computed for categorical variables; while the mean, SD, and range is given for quantitative variable.

|  |  | Completed 5years reviews n (%) or  mean (SD; range) | Completed 10years reviews n (%) or  mean (SD; range) | P-value Test for difference | Authors |
| --- | --- | --- | --- | --- | --- |
| Age at Onset (yrs) | Categories | 36.9(9.2; 18-57) | 36.4(9.4; 18-57) | 0.29 | [8, 10, 11, 19, 40, 41, 54, 55] |
| Sex | Females | 196(77.5) | 196(77.5) | 0.99 |  |
|  | Males | 57(22.5) | 57(22.5) | 0.99 |  |
| Study site | $\mathrm{TAS}$ | 71(31.6) | 64(30.5) | 0.40 | [11, 44] |
|  | $\mathrm{VIC}$ | 55(24.4) | 54(25.8) | 0.37 |  |
|  | $\mathrm{NSW}$ | 34(15.1) | 34(16.2) | 0.36 |  |
|  | $\mathrm{QLD}$ | 65(28.9) | 58(27.6) | 0.38 |  |
| Body mass index ($kg/m^{2}$) | Obese | 84(37.3) | 59(28.1) | 0.02 | *[13, 15, 40, 44, 56]* |
|  | Overweight | 60(26.7) | 77(36.7) | 0.02 |  |
|  | Normal | 81(36.0) | 74(35.2) | 0.44 |  |
| Duration of DMT (DDMT) |  | 1.0(0.8) | 1.1(0.7) | 1.00 | [3] |
| Baseline Number of T2 lesions |  | 8.6(3.4) | 8.6(3.4) | 0.11 | [42, 43, 57, 58] |
| 25(OH) Vitamin D |  | 76.7(28.6, 8.9-192.2) | 78.7(28.6;20.8-192.2) | 0.12 | [6, 12, 13] |
| Smoke tobacco | Yes | 135(60.0) | 124(59.0) | 0.42 | [12, 13, 40, 54] |
|  | No | 90(40.0) | 86(41.0) | 0.42 |  |
| Smoke marijuana | Yes | 8(3.6) | 7(3.3) | 0.44 | [59] |
|  | No | 217(96.4) | 203(96.7) | 0.44 |  |
| Educational level | HE | 57(25.3) | 54(25.7) | 0.46 | NI |
|  | SE | 113(50.2) | 109(51.9) | 0.37 |  |
|  | LSE | 55(24.4) | 47(22.4) | 0.31 |  |
| Relapse ? | Yes | 51(22.7) | 30(14.3) | 0.01 | [60] |
|  | No | 174(77.3) | 180(85.7) | 0.01 |  |
| Worsening of disease ? | Yes | 50(22.2) | 32(15.2) | 0.03 | [1, 2, 4, 33] |
|  | No | 175(77.8) | 178(84.8) | 0.03 |  |
| Relapse counts | 0-2 | 46(20.4) | 9(4.3) | <0.01 | [19, 22, 44, 61] |
|  | 3-4 | 17(7.6) | 0(0.0) | <0.01 |  |
|  | 5-8 | 15(6.7) | 0(0.0) | <0.01 |  |
| Converted to CDMS? | Yes | 178(79.1) | 176(87.6) | 0.01 | [19, 54] |
|  | No | 47(20.9) | 34(16.2) | 0.11 |  |
| Recent immunisation | Yes | 106(47.1) | 0(0.0) | <0.01 | NI |
|  | No | 119(58.9) | 210(100.0) | <0.01 |  |
| Vitamin D supplements | Yes | 53(23.6) | 19(9.0) | <0.01 | [9, 11-15, 22, 44] |
|  | No | 172(76.4) | 191(91.0) | <0.01 |  |
| HADS (Depression score) |  | 10.0(6.8; 0-28) | 10.6(6.3; 0-38) | 0.17 | NI |
| Job change | Yes | 66(29.3) | 49(23.3) | 0.06 | [40] |
|  | No | 159(70.7) | 161(76.7) | 0.08 |  |
| Employment status | FT | 32(14.2) | 32(15.2) | 0.39 | NI |
|  | DP | 22(9.8) | 22(10.5) | 0.41 |  |
|  | WH 13(5.8) | | 13(6.2) | 0.43 |  |
|  | PT | 31(13.8) | 31(14.8) | 0.38 |  |
|  | UE | 127(56.4) | 112(53.3) | 0.17 |  |
| $\Delta$ in sunlight exposure (hrs) (winter-summer) |  | 0.5(1.1; -4.0-4.0) | 0.5(1.1; -40-4.0) | 0.50 | [44, 54] |
| Income levels | $1500-$2000 | 29(12.9) | 29(13.8) | 0.20 | [40] |
|  | $600-$1499 | 79(35.1) | 73(34.8) | 0.24 |  |
|  | $1-$599 | 90(40.0) | 82(39.0) | 0.42 |  |
|  | $0(others) | 27(12.0) | 26(12.4) | 0.45 |  |
| Inter-attack Intervals (yrs) |  | 0.6(0.4; 0-2.0) | 0.8(0.3; 0.2-1.7) | <0.01 | [19] |
| **NI**: Newly Investigated; **FT**=Full-time; **DP**=Disability pension; **WH** =working from home; **PT**=Part-time; **EU**=Unemployed; **CDMS**: Clinically definite MS. $\mathbf{HE=}$ higher education; $\mathbf{SE=}$ secondary education; $\mathbf{LSE=}$ less than secondary education. | | | | | |

|  |  | **Worsening of Disease**  $\boldsymbol{N=2858;}$  $\mathcal{D}\boldsymbol{=1011}$ | | | **Relapses**  $\boldsymbol{N=2858;}$  $\mathcal{D}\boldsymbol{=564}$ | | | **Relapse and/or**  **Worsening of Disease**  $\boldsymbol{N=2858;}\mathcal{D}\boldsymbol{=1377}$ | | |
| --- | --- | --- | --- | --- | --- | --- | --- | --- | --- | --- |
| **Clinical Variables** | **Categories** | $\boldsymbol{\beta}$ | **SE** | **P** | $\boldsymbol{\beta}$ | **SE** | **P** | $\boldsymbol{\beta}$ | **SE** | **P** |
| **Baseline Predictors** | | | | | | | | | | |
| Age at FDE (yrs) |  | 0.01 | 0.01 | 0.16 | -0.01 | 0.01 | 0.01 | -0.01 | 0.01 | 0.86 |
| Sex | Female | -0.14 | 0.10 | 0.16 | 0.05 | 0.12 | 0.69 | -0.11 | 0.08 | 0.16 |
| Study site | $\mathrm{TAS}$ | 0.06 | 0.13 | 0.65 | -0.31 | 0.13 | 0.02 | -0.06 | 0.09 | 0.55 |
|  | $\mathrm{VIC}$ | -0.10 | 0.14 | 0.49 | 0.04 | 0.12 | 0.75 | -0.15 | 0.08 | 0.15 |
|  | $\mathrm{NSW}$ | 0.01 | 0.16 | 0.94 | 0.11 | 0.14 | 0.41 | 0.09 | 0.09 | 0.33 |
|  | $\mathrm{QLD}$ | reference | | | reference | | | reference | | |
| 25(OH)D (nmol/L) | | <-0.01 | <0.01 | 0.05 | <-0.01 | <0.01 | 0.17 | <-0.01 | <0.01 | <0.01 |
| Smoke tobacco | Yes | -0.12 | 0.10 | 0.24 | -0.15 | 0.10 | 0.15 | -0.08 | 0.07 | 0.25 |
| Smoke marijuana | Yes | 0.21 | 0.22 | 0.34 | -0.42 | 0.38 | 0.26 | 0.09 | 0.19 | 0.64 |
| Educational level | HE | -0.11 | 0.12 | 0.35 | -0.01 | 0.15 | 0.97 | -0.08 | 0.08 | 0.40 |
|  | SE | -0.15 | 0.10 | 0.13 | -0.06 | 0.13 | 0.63 | -0.18 | 0.09 | 0.04 |
|  | LSE | reference | | | reference | | | reference | | |
| Number of T2 lesions |  | 0.44 | 0.10 | <0.01 | 0.28 | 0.07 | <0.01 | 0.27 | 0.06 | <0.01 |
| Duration of DMT |  | -0.05 | 0.19 | 0.78 | -0.01 | 0.16 | 0.95 | -0.01 | 0.06 | 0.93 |
| **Time-Dependent Predictors** | | | | | | | | | | |
| RRE | Yes | 0.11 | 0.11 | 0.34 | - | - | - | - | - | - |
| WoD | Yes | - | - | - | 0.47 | 0.11 | <0.01 | - | - | - |
| Body mass index (kg/m^2^) | | 1.25 | 0.40 | <0.01 | 0.63 | 0.39 | 0.10 | 2.30 | 0.99 | 0.02 |
| Number of relapses |  | 0.40 | 0.07 | <0.01 | 0.60 | 0.07 | <0.01 | 0.66 | 0.05 | <0.01 |
| Recent immunisation Yes | | 0.22 | 0.09 | 0.01 | -0.22 | 0.14 | 0.12 | 0.12 | 0.08 | 0.15 |
| Vitamin D supplements | Yes | -1.91 | 0.21 | <0.01 | -0.81 | 0.38 | 0.03 | -0.62 | 0.18 | <0.01 |
| HADS |  | 0.28 | 0.09 | <0.01 | 0.87 | 0.12 | <0.01 | 0.59 | 0.07 | <0.01 |
| Job change | Yes | 0.08 | 0.11 | 0.46 | 0.11 | 0.11 | 0.30 | 0.07 | 0.07 | 0.29 |
| Employment status | FT | -0.17 | 0.15 | 0.24 | -0.11 | 0.14 | 0.43 | -0.26 | 0.11 | 0.02 |
|  | DP | -0.25 | 0.17 | 0.15 | -0.03 | 0.16 | 0.85 | -0.28 | 0.13 | 0.02 |
|  | PT/WH | <0.01 | 0.13 | 0.98 | -0.23 | 0.15 | 0.12 | 0.03 | 0.09 | 0.74 |
|  | UE | reference | | | reference | | | reference | | |
| $\Delta$ sunlight exposure (hrs) | | 0.09 | 0.04 | 0.01 | 0.02 | 0.04 | 0.58 | 0.07 | 0.03 | 0.01 |
| Income levels | $1500-$2000 | 0.29 | 0.23 | 0.21 | 0.81 | 0.19 | <0.01 | 0.46 | 0.17 | 0.01 |
|  | $600-$1499 | 0.60 | 0.15 | <0.01 | 1.03 | 0.16 | <0.01 | 0.63 | 0.12 | <0.01 |
|  | $1-$599 | 0.33 | 0.16 | 0.03 | 0.93 | 0.15 | <0.01 | 0.47 | 0.12 | <0.01 |
|  | $0 | reference | | | reference | | | reference | | |
| log(Inter-attack intervals) | | -0.93 | 0.08 | <0.01 | -0.98 | 0.05 | <0.01 | -3.60 | 0.21 | <0.01 |
| **NB:** -The actual values for P-values <0.01 range between ${10}^{-3}\mathrm{to}{10}^{-12}$.  $\mathcal{D}\boldsymbol{=}$ number of events**,** $\boldsymbol{N=}$number of observations.  **HE**=higher education; **SE**=secondary education; **LSE**=less than secondary education; **FT**=Full time; Disability pension**; PT/WH=**Part-time/work from home; **UE** =Unemployed. **HADS**: Hospital anxiety depression score.  **NSW**: New South Wales. **QLD**: Queensland. **TAS**: Tasmania. **VIC**: Victoria;  **Inter-attack intervals**: difference between event stop and event start time.  $\boldsymbol{\Delta}$ **in sunlight exposure**: Change in hours of sunlight exposure (winter-summer).  **25(OH)D**: 25 hydroxy vitamin D levels measured in units of nmol/L  **FDE**: First demyelinating event. | | | | | | | | | | |

**Supplementary Table 2**. Crude Cox Model: Regression coefficients ($\beta$), standard errors (SE), and p-values (Pr) possible clinical predictors for risk of worsening of disease (WoD), recurring relapsing events (RRE), and relapse and/or worsening of disease (RWoD).

**Supplementary Table 3.** Multivariate Cox models : Adjusted regression coefficients **(**$\boldsymbol{\beta}$), standard errors (**SE**), and p-values (**P**) for genetic predictors (SNPs) included in the genetic prognostic index (GPI) when predicting the risk of worsening of disease (**WoD**), recurrent relapsing events (**RRE**), and relapse and/or worsening of disease (**RWoD**).

| **rsID** | **Chr:Pos** | **GeneID** | *β* | **SE** | **P** |
| --- | --- | --- | --- | --- | --- |
| **Worsening of Disease**  $\boldsymbol{N=2858;}\mathcal{D}\boldsymbol{=1011}$ | | | | | |
| rs1177228 | 2:61242410 | PUS10*\|*KIAA1841 | -2.44 | 0.41 | 1.85*×*10*^−^*^9^ |
| rs1177228^ǂ^ | 2:61242410 | PUS10*\|*KIAA1841 | 0.05 | 0.01 | 6.31*×*10*^−^*^4^ |
| rs1177228*^*^* | 2:61242410 | PUS10*\|*KIAA1841 | -0.44 | 0.13 | 1.67*×*10*^−^*^2^ |
| rs962052 ^ǂ^ | 2:151644203 | RBM43*\|*RND3 | -0.24 | 0.08 | 1.49*×*10*^−^*^6^ |
| rs9878602 | 3:71535338 | FOXP1 | -1.30 | 0.39 | 8.13*×*10*^−^*^4^ |
| rs9878602^ǂ^ | 3:71535338 | FOXP1 | 0.04 | 0.01 | 2.73*×*10*^−^*^4^ |
| rs2331964*^*^* | 3:121542898 | FBXO40*\|*HCLS1 | 0.85 | 0.45 | 5.75*×*10*^−^*^2^ |
| rs2331964^ǂ^ | 3:121542898 | FBXO40*\|*HCLS1 | -0.02 | 0.01 | 1.21*×*10*^−^*^1^ |
| rs6837324 | 4:48127262 | NIPAL1*\|*TXK | 0.13 | 0.07 | 4.12*×*10*^−^*^2^ |
| rs17051321^ǂ^ | 4:122119449 | TNIP3*\|*NDNF | *<*-0.01 | *<*0.01 | 6.19*×*10*^−^*^2^ |
| rs7731626 | 5:55444683 | ANKRD55 | -0.15 | 0.07 | 3.37*×*10*^−^*^2^ |
| rs3129889 | 6:32413545 | HLA-DRB1 | -0.11 | 0.40 | 7.9*×*10*^−^*^1^ |
| rs3129889*^*^* | 6:32413545 | HLA-DRB1 | 0.10 | 0.14 | 4.45*×*10*^−^*^1^ |
| rs3129889^ǂ^ | 6:32413545 | HLA-DRB1 | *<*0.01 | 0.01 | 8.85*×*10*^−^*^1^ |
| rs3819292 | 6:31322522 | HLA-B*\|*MIR6891 | 1.57 | 0.47 | 8.33*×*10*^−^*^4^ |
| rs3819292^ǂ^ | 6:31322522 | HLA-B*\|*MIR6891 | -0.05 | 0.01 | 4.51*×*10*^−^*^4^ |
| rs62420820^ǂ^ | 6:137438057 | IL20RA*\|*IL22RA2 | *<*-0.01 | *<*0.01 | 1.55*×*10*^−^*^1^ |
| rs9266629^ǂ^ | 6:31346822 | ZDHHC20P2*\|*FGFR3P1 | *<*0.01 | *<*0.01 | 6.93*×*10*^−^*^2^ |
| rs11751659^ǂ^ | 6:33081632 | HLA-DPB2 | 0.01 | *<*0.01 | 4.77*×*10*^−^*^3^ |
| rs3097671 | 6:33047612 | tagging(HLA-DPB1*0501) | 0.34 | 0.13 | 1.02*×*10*^−^*^2^ |
| rs3097671*^*^* | 6:33047612 | tagging(HLA-DPB1*0501) | 0.20 | 0.16 | 2.07*×*10*^−^*^1^ |
| rs10951154 | 7:27135314 | HOXA1*\|*SKAP2 | 1.07 | 0.63 | 9.17*×*10*^−^*^2^ |
| rs10951154^ǂ^ | 7:27135314 | HOXA1*\|*SKAP2 | -0.03 | 0.02 | 6.29*×*10*^−^*^2^ |
| rs4728142*^*^* | 7:128573967 | IRF5*\|*TNPO3 | -0.16 | 0.07 | 2.43*×*10*^−^*^2^ |
| rs3923387 | 8:144986793 | PLEC*\|*PARP10 | -1.05 | 0.40 | 8.91*×*10*^−^*^3^ |
| rs3923387^ǂ^ | 8:144986793 | PLEC*\|*PARP10 | 0.03 | 0.01 | 1.16*×*10*^−^*^2^ |
| rs61863928 | 10:64449549 | ADO | 0.85 | 0.40 | 3.52*×*10*^−^*^2^ |
| rs61863928^ǂ^ | 10:64449549 | ADO | -0.03 | 0.01 | 2.95*×*10*^−^*^3^ |
| rs61863928*^*^* | 10:64449549 | ADO | -0.38 | 0.11 | 8.30*×*10*^−^*^4^ |
| rs17741873 | 10:75653800 | ECD*\|*FAM149B1 | 0.44 | 0.14 | 1.54*×*10*^−^*^3^ |
| rs17741873*^*^* | 10:75653800 | ECD*\|*FAM149B1 | 0.32 | 0.16 | 4.80*×*10*^−^*^2^ |
| rs1112718 | 10:94479107 | IDE*\|*KIF11 | 0.98 | 0.44 | 2.48*×*10*^−^*^2^ |
| rs1112718^ǂ^ | 10:94479107 | IDE*\|*KIF11 | -0.03 | 0.01 | 1.30*×*10*^−^*^2^ |
| rs4409785 | 11:95311422 | SESN3 | -2.74 | 0.56 | 1.20*×*10*^−^*^6^ |
| rs4409785*^*^* | 11:95311422 | SESN3 | -0.34 | 0.16 | 3.46*×*10*^−^*^2^ |
| rs4409785^ǂ^ | 11:95311422 | SESN3 | 0.06 | 0.02 | 5.36*×*10*^−^*^5^ |
| rs3184504 | 12:111884608 | SH2B3*\|*CUX2 | 1.34 | 0.49 | 5.75*×*10*^−^*^3^ |
| rs3184504^ǂ^ | 12:111884608 | SH2B3*\|*CUX2 | -0.03 | 0.01 | 1.02*×*10*^−^*^2^ |
| rs2286974 | 16:11114512 | CLEC16A | -0.14 | 0.07 | 2.91*×*10*^−^*^2^ |
| rs9955954 | 18:56348044 | MALT1*\|*LOC101927322 | -1.72 | 0.45 | 1.19*×*10*^−^*^4^ |
| rs9955954^ǂ^ | 18:56348044 | MALT1*\|*LOC101927322  4 | 0.04 | 0.01 | 1.06*×*10*^−^*^3^ |
| rs11083862 | 19:47638539 | NPAS1*\|*TMEM160 | 0.18 | 0.07 | 9.82*×*10*^−^*^3^ |
| rs140522 | 22:50971266 | LMF2*\|*SCO2 | -0.14 | 0.07 | 4.75*×*10*^−^*^2^ |
| Relapses  $N=2858; \mathcal{D}=564$ | | | | | |
| rs11809700 | 1:93152635 | EVI5*\|*FAM69A | -0.38 | 0.10 | 9.43*×*10*^−^*^5^ |
| rs2317231 | 1:157686337 | FCRL5*\|*FCRL4 | -0.19 | 0.08 | 1.53*×*10*^−^*^2^ |
| rs57116599 | 2:112770799 | MERTK*\|*TMEM87B | 0.21 | 0.10 | 4.62*×*10*^−^*^2^ |
| rs13136820 | 4:40307564 | CHRNA9 | 1.11 | 0.50 | 2.62*×*10*^−^*^2^ |
| rs7855251 | 9:100868189 | NANS*\|*TRIM14 | -1.86 | 0.56 | 8.83*×*10*^−^*^4^ |
| rs1087056 | 10:31395761 | ZEB1*\|*LOC100505502 | 1.79 | 0.48 | 2.17*×*10*^−^*^4^ |
| rs17741873 | 10:75653800 | ECD*\|*FAM149B1 | -1.77 | 0.58 | 2.19*×*10*^−^*^3^ |
| rs1112718 | 10:94479107 | IDE*\|*KIF11 | 0.20 | 0.08 | 8.01*×*10*^−^*^3^ |
| rs11231749 | 11:64095178 | FLRT1*\|*VEGFB | 0.29 | 0.09 | 7.52*×*10*^−^*^4^ |
| rs4262739 | 11:128421175 | ETS1 | 0.21 | 0.10 | 2.63*×*10*^−^*^2^ |
| rs12147246 | 14:103265844 | TRAF3 | -2.13 | 0.50 | 1.82*×*10*^−^*^5^ |
| rs2248137 | 20:52789743 | CYP24A1 | 0.28 | 0.08 | 6.87*×*10*^−^*^4^ |
| rs2844482 | 6:31539767 | LST1(class III haplotype) | 1.75 | 0.47 | 1.72*×*10*^−^*^4^ |
| rs10093 | 6:32609173 | HLA-DQA1 | 0.21 | 0.11 | 5.67*×*10*^−^*^2^ |
| rs3135024 | 6:33047466 | HLA-DPA1*\|*HLA-DPB1 | -0.18 | 0.09 | 4.27*×*10*^−^*^2^ |
| rs11751659 | 6:33081632 | HLA-DPB2 | 0.89 | 0.78 | 2.52*×*10*^−^*^1^ |
| rs9277626 | 6:33081823 | HLA-DPB2 | 0.39 | 0.12 | 9.30*×*10*^−^*^4^ |
| rs3129889 | 6:32413545 | HLA-DRB1 | 0.17 | 0.60 | 7.82*×*10*^−^*^1^ |
| rs3737798*^*^* | 1:160389984 | COPA*\|*VANGL2 | 0.24 | 0.06 | 3.48*×*10*^−^*^5^ |
| rs11899404*^*^* | 2:12607893 | LOC100506457 | -0.36 | 0.07 | 6.66*×*10*^−^*^8^ |
| rs9992763^ǂ^ | 4:109058718 | LEF1*\|*RPL34-AS1 | -0.01 | *<*0.01 | 1.55*×*10*^−^*^4^ |
| rs17051321^ǂ^ | 4:122119449 | TNIP3*\|*NDNF | 0.01 | *<*0.01 | 3.36*×*10*^−^*^4^ |
| rs7731626 *^*^* | 5:55444683 | ANKRD55 | 0.17 | 0.07 | 1.28*×*10*^−^*^2^ |
| rs12211604*^*^* | 6:7100029 | TRAF3 | -0.11 | 0.06 | 6.23*×*10*^−^*^2^ |
| rs719316^ǂ^ | 6:16672760 | ATXN1 | -0.01 | *<*0.01 | 4.02*×*10*^−^*^2^ |
| rs802730*^*^* | 6:128280104 | THEMIS*\|*PTPRK | -0.18 | 0.08 | 1.82*×*10*^−^*^2^ |
| rs62420820*^*^* | 6:137438057 | IL20RA*\|*IL22RA2 | 0.53 | 0.08 | 1.78*×*10*^−^*^10^ |
| rs1800693*^*^* | 12:6440009 | PLEKHG6*\|*TNFRSF1A | -0.11 | 0.07 | 1.27*×*10*^−^*^1^ |
| rs3184504*^*^* | 12:111884608 | SH2B3*\|*CUX2 | 0.13 | 0.06 | 2.43*×*10*^−^*^2^ |
| rs2286974 | 16:11213951 | DEXI*\|*CLEC16A | 0.01 | *<*0.01 | 2.87*×*10*^−^*^3^ |
| rs28834106^ǂ^ | 19:10592144 | TYK2*\|*PDE4A | -0.01 | *<*0.01 | 1.14*×*10*^−^*^3^ |
| rs4808760*^*^* | 19:18301979 | IL12RB1*\|*MAST3 | 0.24 | 0.07 | 2.12*×*10*^−^*^4^ |
| rs760517*^*^* | 22:37258986 | NCF4 | -0.19 | 0.05 | 6.66*×*10*^−^*^4^ |
| rs114071505*^*^* | 6:30060631 | RNF39*\|*TRIM31 | -0.32 | 0.19 | 9.18*×*10*^−^*^2^ |
| rs3093982*^*^* | 6:31497244 | MCCD1 | -0.52 | 0.09 | 2.39*×*10*^−^*^8^ |
| rs3819292*^*^* | 6:31518354 | NFKBIL1 | 0.56 | 0.09 | 1.03*×*10*^−^*^10^ |
| rs9271366^ǂ^ | 6:32586854 | DBR1*\|*DQA1 | 0.02 | 0.01 | 8.32*×*10*^−^*^2^ |
| rs1087056^ǂ^ | 10:31395761 | ZEB1*\|*LOC100505502 | -0.05 | 0.01 | 6.99*×*10*^−^*^4^ |
| rs13136820^ǂ^ | 4:40307564 | CHRNA9 | -0.03 | 0.01 | 4.91*×*10*^−^*^2^ |
| rs7855251^ǂ^ | 9:100868189 | NANS*\|*TRIM14 | 0.05 | 0.02 | 8.00*×*10*^−^*^4^ |
| rs17741873^ǂ^ | 10:75653800 | ECD*\|*FAM149B1 | 0.05 | 0.02 | 4.24*×*10*^−^*^3^ |
| rs12147246^ǂ^ | 14:103265844 | TRAF3 | 0.05 | 0.01 | 1.75*×*10*^−^*^4^ |
| rs2844482^ǂ^ | 6:31539767 | LST1(class III haplotype) | -0.05 | 0.01 | 1.05*×*10*^−^*^4^ |
| rs11751659^ǂ^ | 6:33081632 | HLA-DPB2 | -0.03 | 0.02 | 1.69*×*10*^−^*^1^ |
| rs9992763*^*^* | 4:109058718 | LEF1*\|*RPL34-AS1 | -0.24 | 0.09 | 9.62*×*10*^−^*^3^ |
| rs719316*^*^* | 6:16672760 | ATXN1 | 0.28 | 0.07 | 1.02*×*10*^−^*^4^ |
| rs7855251*^*^* | 9:100868189 | NANS*\|*TRIM14 | -0.26 | 0.09 | 3.89*×*10*^−^*^3^ |
| rs3129889*^*^* | 6:32413545 | HLA-DRB1 | 0.12 | 0.11 | 2.74*×*10*^−^*^1^ |
| rs3129889^ǂ^ | 6:32413545 | HLA-DRB1 | -0.01 | 0.02 | 5.55*×*10*^−^*^1^ |
| Relapse and/or Worsening of Disease  $N=2858; \mathcal{D}=1377$ | | | | | |
| chr1:32738415 | 1:32738415 | FAM167B/LCK | -0.14 | 0.05 | 1.23*×*10*^−^*^2^ |
| rs11809700 | 1:93152635 | EVI5*\|*FAM69A | -0.06 | 0.05 | 2.82*×*10*^−^*^1^ |
| rs34723276 | 1:101290432 | SLC30A7 | -0.07 | 0.05 | 1.65*×*10*^−^*^1^ |
| rs2317231 | 1:157686337 | FCRL5*\|*FCRL4 | -0.26 | 0.05 | 6.53*×*10*^−^*^7^ |
| rs59655222 | 1:200875897 | C1orf106*\|*CACNA1S | 0.90 | 0.31 | 3.90*×*10*^−^*^3^ |
| rs11899404 | 2:12607893 | LOC100506457 | -1.36 | 0.35 | 1.06*×*10*^−^*^4^ |
| rs12622670 | 2:68646536 | PLEK | 0.38 | 0.32 | 2.27*×*10*^−^*^1^ |
| rs9878602 | 3:71535338 | FOXP1 | -0.88 | 0.28 | 1.75*×*10*^−^*^3^ |
| rs4325907 | 3:101749022 | ZPLD1 | 0.15 | 0.05 | 3.26*×*10*^−^*^3^ |
| rs6837324 | 4:48127262 | NIPAL1*\|*TXK | 1.16 | 0.31 | 1.66*×*10*^−^*^4^ |
| rs9992763 | 4:109058718 | LEF1*\|*RPL34-AS1 | 0.89 | 0.26 | 7.66*×*10*^−^*^4^ |
| rs17051321 | 4:122119449 | TNIP3*\|*NDNF | 0.09 | 0.05 | 8.77*×*10*^−^*^2^ |
| rs34681760 | 5:6712834 | PAPD7 | -1.00 | 0.31 | 1.13*×*10*^−^*^3^ |
| rs11749040 | 5:40396425 | TTC33*\|*PRKAA1 | -0.12 | 0.05 | 9.02*×*10*^−^*^3^ |
| rs244656 | 5:133449827 | CDKN2AIPNL | 0.20 | 0.06 | 5.69*×*10*^−^*^2^ |
| rs249677 | 5:141539339 | NDFIP1 | -0.27 | 0.06 | 1.24*×*10*^−^*^6^ |
| rs67111717 | 5:176790162 | MXD3*\|*RGS14 | 0.49 | 0.32 | 1.31*×*10*^−^*^2^ |
| rs2327586 | 6:135495226 | HBS1L | -0.88 | 0.29 | 2.73*×*10*^−^*^3^ |
| rs10951154 | 7:27135314 | HOXA1*\|*SKAP2 | 0.70 | 0.29 | 1.66*×*10*^−^*^2^ |
| rs12722559 | 10:6070273 | IL15RA*\|*IL2RA | -1.58 | 0.35 | 5.68*×*10*^−^*^6^ |
| rs1087056 | 10:31395761 | ZEB1*\|*LOC100505502 | -0.22 | 0.06 | 1.92*×*10*^−^*^4^ |
| rs61708525 | 12:94661453 | PLXNC1*\|*CEP83 | 1.15 | 0.35 | 1.06*×*10*^−^*^3^ |
| rs3184504 | 12:111884608 | SH2B3*\|*CUX2 | 0.48 | 0.29 | 9.74*×*10*^−^*^2^ |
| rs6496663 | 15:90887584 | IQGAP1 | -0.41 | 0.28 | 1.42*×*10*^−^*^1^ |
| rs2286974 | 16:11213951 | DEXI*\|*CLEC16A | -0.78 | 0.33 | 1.79*×*10*^−^*^2^ |
| rs4796224 | 17:34842521 | MYO19*\|*GGNBP2 | -0.06 | 0.05 | 1.69*×*10*^−^*^1^ |
| rs883871 | 17:38252660 | NR1D1*\|*IKZF3 | -0.46 | 0.25 | 6.16*×*10*^−^*^2^ |
| rs11079784 | 17:45702280 | ITGB3*\|*EFCAB13 | -0.82 | 0.31 | 7.73*×*10*^−^*^3^ |
| rs4812772 | 20:42579051 | TOX2 | -0.17 | 0.06 | 3.13*×*10*^−^*^3^ |
| rs9610458 | 22:22205353 | PPM1F*\|*TOP3B | 0.48 | 0.28 | 8.52*×*10*^−^*^2^ |
| rs5756405 | 22:37310954 | NCF4 | -0.95 | 0.31 | 2.32*×*10*^−^*^3^ |
| rs137955 | 22:40291807 | ENTHD1*\|*GRAP2 | 0.72 | 0.31 | 2.06*×*10*^−^*^2^ |
| rs3129898 | 6:32422125 | HLA-DRA | 1.85 | 0.62 | 2.74*×*10*^−^*^3^ |
| rs4081559 | 6:31323677 | HLA-B | 0.94 | 0.34 | 5.22*×*10*^−^*^3^ |
| rs2844482 | 6:31539767 | LST1(class III haplotype) | 0.76 | 0.29 | 9.01*×*10*^−^*^3^ |
| rs9271366 | 6:32586854 | DBR1*\|*DQA1 | -2.38 | 1.28 | 6.32*×*10*^−^*^2^ |
| rs3097671 | 6:33047612 | tagging(HLA-DPB1*0501) | 0.11 | 0.30 | 6.99*×*10*^−^*^1^ |
| rs9277626 | 6:33081823 | HLA-DPB2 | 0.33 | 0.32 | 2.97*×*10*^−^*^1^ |
| rs3129889 | 6:32413545 | HLA-DRB1 | 1.08 | 1.14 | 3.44*×*10*^−^*^1^ |
| rs6738544^ǂ^ | 2:191989356 | STAT4 | *<*-0.01 | *<*0.01 | 3.46*×*10*^−^*^2^ |
| rs11919880^ǂ^ | 3:32962051 | CCR4 | *<*-0.01 | *<*0.01 | 2.97*×*10*^−^*^2^ |
| rs9843355 | 3:119228508 | ARHGAP31*\|*TMEM39A | *<*0.01 | *<*0.01 | 5.65*×*10*^−^*^2^ |
| rs1014486*^*^* | 3:159691112 | IL12A-AS1*\|*IQCJ-SCHIP1-AS1 | -0.26 | 0.05 | 2.58*×*10*^−^*^8^ |
| rs2705616^ǂ^ | 4:87862396 | PTPN13*\|*SLC10A6 | *<*0.01 | *<*0.01 | 2.24*×*10*^−^*^1^ |
| rs6533052^ǂ^ | 4:103911781 | MANBA*\|*SLC9B2 | *<*-0.01 | *<*0.01 | 4.55*×*10*^−^*^3^ |
| rs2726479*^*^* | 4:106255589 | TET2*\|*PPA2 | -0.24 | 0.05 | 4.96*×*10*^−^*^7^ |
| rs10063294^ǂ^ | 5:35877505 | IL7R | *<*0.01 | *<*0.01 | 6.82*×*10*^−^*^2^ |
| rs3819292*^*^* | 6:31322522 | HLA-B*\|*MIR6891 | 0.16 | 0.06 | 5.47*×*10*^−^*^3^ |
| rs2229092^ǂ^ | 6:31540757 | LTA | *<*-0.01 | *<*0.01 | 1.41*×*10*^−^*^1^ |
| rs3135024^ǂ^ | 6:33047466 | HLA-DPA1*\|*HLA-DPB1 | -0.01 | *<*0.01 | 2.99*×*10*^−^*^3^ |
| rs735542^ǂ^ | 8:128175696 | PCAT2*\|*PRNCR1 | *<*0.01 | *<*0.01 | 1.51*×*10*^−^*^1^ |
| rs1112718 *^*^* | 10:94479107 | IDE*\|*KIF11 | -0.14 | 0.04 | 6.08*×*10*^−^*^4^ |
| rs1365120*^*^* | 11:36438075 | PRR5L*\|*TRAF6 | 0.11 | 0.04 | 1.03*×*10*^−^*^2^ |
| rs4409785^ǂ^ | 11:95311422 | SESN3 | *<*-0.01 | *<*0.01 | 2.06*×*10*^−^*^2^ |
| rs1800693^ǂ^ | 12:6440009 | PLEKHG6*\|*TNFRSF1A | *<*-0.01 | *<*0.01 | 1.28*×*10*^−^*^1^ |
| rs11852059^ǂ^ | 14:52306091 | GNG2 | *<*-0.01 | *<*0.01 | 8.67*×*10*^−^*^2^ |
| rs34947566*^*^* | 16:11412926 | TNP2*\|*PRM2 | 0.13 | 0.04 | 2.60*×*10*^−^*^3^ |
| rs35703946*^*^* | 16:86021505 | IRF8*\|*LINC01082 | 0.05 | 0.05 | 2.66*×*10*^−^*^1^ |
| rs4940730*^*^* | 18:56269737 | ALPK2 | -0.17 | 0.05 | 3.22*×*10*^−^*^4^ |
| rs2469434^ǂ^ | 18:67544046 | CD226 | *<*-0.01 | *<*0.01 | 2.81*×*10*^−^*^1^ |
| rs28834106*^*^* | 19:10592144 | TYK2*\|*PDE4A | -0.12 | 0.05 | 1.61*×*10*^−^*^2^ |
| rs6742^ǂ^ | 20:62374441 | RTEL1*\|*TNFRSF6B | *<*0.01 | *<*0.01 | 5.98*×*10*^−^*^2^ |
| rs140522^ǂ^ | 22:50971266 | LMF2*\|*SCO2 | *<*-0.01 | *<*0.01 | 1.32*×*10*^−^*^2^ |
| rs3129889^ǂ^ | 6:32413545 | HLA-DRB1 | *<*0.01 | 0.03 | 9.07*×*10*^−^*^1^ |
| rs3093982*^*^* | 6:31497244 | MCCD1 | -0.14 | 0.06 | 2.62*×*10*^−^*^2^ |
| rs3819292^ǂ^ | 6:31518354 | NFKBIL1 | -0.01 | *<*0.01 | 9.27*×*10*^−^*^4^ |
| rs10093^ǂ^ | 6:32609173 | HLA-DQA1 | 0.01 | *<*0.01 | 2.00*×*10*^−^*^5^ |
| rs11751659*^*^* | 6:33081632 | HLA-DPB2 | 0.10 | 0.05 | 6.89*×*10*^−^*^2^ |
| rs1087056*^*^* | 10:31395761 | ZEB1*\|*LOC100505502 | -0.15 | 0.06 | 8.61*×*10*^−^*^3^ |
| rs12722559^ǂ^ | 10:6070273 | IL15RA*\|*IL2RA | 0.05 | 0.01 | 5.64*×*10*^−^*^7^ |
| rs59655222^ǂ^ | 1:200875897 | C1orf106*\|*CACNA1S | -0.03 | 0.01 | 3.80*×*10*^−^*^3^ |
| rs11899404^ǂ^ | 2:12607893 | LOC100506457 | 0.03 | 0.01 | 3.42*×*10*^−^*^4^ |
| rs12622670^ǂ^ | 2:68646536 | PLEK | -0.01 | 0.01 | 2.02*×*10*^−^*^1^ |
| rs9878602^ǂ^ | 3:71535338 | FOXP1 | 0.02 | 0.01 | 2.36*×*10*^−^*^3^ |
| rs6837324 ^ǂ^ | 4:48127262 | NIPAL1*\|*TXK | -0.03 | 0.01 | 8.52*×*10*^−^*^4^ |
| rs9992763^ǂ^ | 4:109058718 | LEF1*\|*RPL34-AS1 | -0.02 | 0.01 | 9.96*×*10*^−^*^4^ |
| rs34681760^ǂ^ | 5:6712834 | PAPD7 | 0.03 | 0.01 | 7.01*×*10*^−^*^4^ |
| rs67111717^ǂ^ | 5:176790162 | MXD3*\|*RGS14 | -0.02 | 0.01 | 9.10*×*10*^−^*^2^ |
| rs2327586^ǂ^ | 6:135495226 | HBS1L | 0.02 | 0.01 | 1.48*×*10*^−^*^2^ |
| rs10951154^ǂ^ | 7:27135314 | HOXA1*\|*SKAP2 | -0.02 | 0.01 | 1.35*×*10*^−^*^2^ |
| rs61708525^ǂ^ | 12:94661453 | PLXNC1*\|*CEP83 | -0.03 | 0.01 | 1.54*×*10*^−^*^3^ |
| rs3184504^ǂ^ | 12:111884608 | SH2B3*\|*CUX2 | -0.01 | 0.01 | 1.03*×*10*^−^*^1^ |
| rs6496663^ǂ^ | 15:90887584 | IQGAP1 | 0.01 | 0.01 | 2.56*×*10*^−^*^1^ |
| rs2286974^ǂ^ | 16:11213951 | DEXI*\|*CLEC16A | 0.02 | 0.01 | 2.04*×*10*^−^*^2^ |
| rs883871^ǂ^ | 17:38252660 | NR1D1*\|*IKZF3 | 0.01 | 0.01 | 9.68*×*10*^−^*^2^ |
| rs11079784^ǂ^ | 17:45702280 | ITGB3*\|*EFCAB13 | 0.02 | 0.01 | 5.19*×*10*^−^*^3^ |
| rs9610458^ǂ^ | 22:22205353 | PPM1F*\|*TOP3B | -0.01 | 0.01 | 1.84*×*10*^−^*^1^ |
| rs5756405^ǂ^ | 22:37310954 | NCF4 | 0.02 | 0.01 | 1.27*×*10*^−^*^2^ |
| rs137955^ǂ^ | 22:40291807 | ENTHD1*\|*GRAP2 | -0.03 | 0.01 | 4.38*×*10*^−^*^3^ |
| rs3129898^ǂ^ | 6:32422125 | HLA-DRA | -0.05 | 0.02 | 6.65*×*10*^−^*^3^ |
| rs4081559^ǂ^ | 6:31323677 | HLA-B | -0.03 | 0.01 | 6.01*×*10*^−^*^3^ |
| rs2844482^ǂ^ | 6:31539767 | LST1(class III haplotype) | -0.02 | 0.01 | 3.03*×*10*^−^*^2^ |
| rs9271366^ǂ^ | 6:32586854 | DBR1*\|*DQA1 | 0.04 | 0.03 | 2.57*×*10*^−^*^1^ |
| rs3097671^ǂ^ | 6:33047612 | tagging(HLA-DPB1*0501) | *<*0.01 | 0.01 | 8.94*×*10*^−^*^1^ |
| rs9277626^ǂ^ | 6:33081823 | HLA-DPB2 | -0.01 | 0.01 | 4.21*×*10*^−^*^1^ |
| rs11899404*^*^* | 2:12607893 | LOC100506457 | -0.09 | 0.06 | 1.55*×*10*^−^*^1^ |
| rs10063294*^*^* | 5:35877505 | IL7R | 0.14 | 0.07 | 3.99*×*10*^−^*^2^ |
| rs244656*^*^* | 5:133449827 | CDKN2AIPNL | 0.21 | 0.06 | 8.56*×*10*^−^*^4^ |
| rs2229092*^*^* | 6:31540757 | LTA | -0.09 | 0.05 | 4.12*×*10*^−^*^2^ |
| rs10951154*^*^* | 7:27135314 | HOXA1*\|*SKAP2 | -0.13 | 0.05 | 1.58*×*10*^−^*^2^ |
| rs735542*^*^* | 8:128175696 | PCAT2*\|*PRNCR1 | -0.12 | 0.07 | 1.15*×*10*^−^*^1^ |
| rs61708525*^*^* | 12:94661453 | PLXNC1*\|*CEP83 | 0.21 | 0.06 | 2.74*×*10*^−^*^4^ |
| rs137955*^*^* | 22:40291807 | ENTHD1*\|*GRAP2 | -0.16 | 0.05 | 4.19*×*10*^−^*^3^ |
| rs3129889*^*^* | 6:32413545 | HLA-DRB1 | 0.16 | 0.06 | 1.18*×*10*^−^*^2^ |
| rs4081559*^*^* | 6:31323677 | HLA-B | -0.34 | 0.06 | 1.55*×*10*^−^*^8^ |
| rs3097671 *^*^* | 6:33047612 | tagging(HLA-DPB1*0501) | -0.12 | 0.06 | 2.42*×*10*^−^*^2^ |
| ***:** Interaction with time.  **ǂ:** Interaction with standardized latitudinal coordinates.  **Note**:  -The regression coefficients were adjusted for the effects of sex, age at first demyelinating event, DMT duration, and number of baseline T2 lesions.  -The SNPs presented on this table were included in the genetic prognostic index (GPI) when predicting the different endpoints. These form the basis of the core or final cox regression models mentioned in the text. | | | | | |

**Supplementary Table 4.** Univariate landmark Supermodel Cox regression on the clinical-env ($\mathbf{CEPI}$), genetic ( $\mathbf{GPI}$), and clinical-env-genotype ($\mathbf{CEGPI}$) cross-validation based prognostic indices across landmark time points $\boldsymbol{(t}_{\boldsymbol{LM}})$*,* running from 0 until 5years with 1 year distance apart, and a prediction window of width $w = 5 years$.

| $\boldsymbol{t}_{\boldsymbol{LM}}$  **(yrs.)** | **# Obs** | **# Events** | **# At**  **Risk** | **CEPI** | | **GPI** | | **CEGPI** | |
| --- | --- | --- | --- | --- | --- | --- | --- | --- | --- |
| **Worsening of disease (WoD)** | | | | | | | | | |
|  |  |  |  | $\boldsymbol{\beta}$ **(SE)** | $\boldsymbol{\chi}^{\boldsymbol{2}}$ | $\boldsymbol{\beta}$**(SE)** | $\boldsymbol{\chi}^{\boldsymbol{2}}$ | $\boldsymbol{\beta}$ **(SE)** | $\boldsymbol{\chi}^{\boldsymbol{2}}$ |
| 0 | 2858 | 840 | 253 | 0.94(0.03) | 806 | 0.78(0.05) | 258 | 0.96(0.03) | 923 |
| 1 | 2487 | 827 | 249 | 0.80(0.04) | 280 | 0.89(0.05) | 290 | 0.90(0.04) | 465 |
| 2 | 2050 | 674 | 236 | 1.27(0.08) | 240 | 0.91(0.06) | 221 | 1.30(0.07) | 393 |
| 3 | 1616 | 479 | 223 | 1.56(0.01) | 232 | 1.14(0.08) | 239 | 1.59(0.08) | 393 |
| 4 | 1204 | 290 | 215 | 1.76(0.13) | 154 | 1.38(0.10) | 207 | 1.85(0.11) | 298 |
| 5 | 766 | 157 | 202 | 1.71(0.20) | 66 | 1.68(0.11) | 160 | 2.01(0.16) | 179 |
| **Relapse (RRE)** | | | | | | | | | |
| 0 | 2858 | 398 | 253 | 0.90(0.03) | 1125 | 0.86(0.04) | 523 | 0.97(0.03) | 1131 |
| 1 | 2496 | 289 | 252 | 0.86(0.04) | 349 | 0.82(0.04) | 296 | 0.92(0.04) | 380 |
| 2 | 2235 | 259 | 247 | 1.03(0.05) | 293 | 0.87(0.05) | 284 | 1.00(0.05) | 314 |
| 3 | 1967 | 236 | 241 | 1.07(0.06) | 270 | 0.90(0.05) | 261 | 1.05(0.06) | 289 |
| 4 | 1692 | 208 | 226 | 1.12(0.06) | 232 | 0.95(0.06) | 238 | 1.09(0.06) | 254 |
| 5 | 1466 | 164 | 222 | 1.24(0.08) | 201 | 1.02(0.07) | 201 | 1.21(0.07) | 220 |
| **Relapse and/or Worsening of disease (RWoD)** | | | | | | | | | |
| 0 | 2858 | 1148 | 253 | 0.91(0.02) | 1557 | 0.83(0.03) | 595 | 0.98(0.02) | 1612 |
| 1 | 2394 | 1009 | 249 | 0.79(0.04) | 477 | 0.71(0.04) | 280 | 0.84(0.03) | 539 |
| 2 | 1987 | 837 | 236 | 1.20(0.06) | 391 | 0.75(0.05) | 238 | 1.06(0.05) | 417 |
| 3 | 1159 | 619 | 225 | 1.42(0.07) | 387 | 0.85(0.06) | 213 | 1.22(0.06) | 401 |
| 4 | 1144 | 389 | 219 | 1.61(0.09) | 323 | 0.96(0.07) | 168 | 1.35(0.07) | 325 |
| 5 | 712 | 220 | 204 | 1.65(0.09) | 196 | 1.00(0.10) | 94 | 1.37(0.09) | 193 |
| $\mathbf{t}_{\mathbf{LM}}=$ Landmark time points in years. $\mathbf{SE}=$ standard errors**, # Events**: number of events.  **# At risk**: number of subjects at risk. **# Obs**: total number of observations;  **CEPI**: Clinical-Env Prognostic Index (Clinical ǂ Environmental predictors)  **GPI:** Genetic Prognostic Index (Cumulative effects of single nucleotide polymorphisms markers)  **CEGPI**: Clinical-Env-Genotypic Prognostic Index = CEPI + GPI (clinical + environmental + genetic) | | | | | | | | | |

**Supplementary Table 5.** Estimated regression coefficients ($\boldsymbol{\beta}$) and standard errors (**SE**) for the stratified and proportional baselines landmark supermodels without (LM-fixed) and with (LM-dependent) linear (s/5) and quadratic $\left( s/5 \right)^{2}$landmark interactions using a prediction window of window $\boldsymbol{w}$**=2.5**years, with a distance of 0.1year. Parameter estimates for the baseline hazards are not shown.

| **Landmark**  **Supermodel** | | **Clinical-Env.** | **Genetic** | **Clinico-**  **genotypic** |
| --- | --- | --- | --- | --- |
|  | **Time**  **Function** | $\boldsymbol{\beta}$**(SE)** | $\boldsymbol{\beta}$**(SE)** | $\boldsymbol{\beta}$**(SE)** |
| **Worsening of disease (**$\boldsymbol{N=2858;}\mathcal{D}\boldsymbol{=1011}$**)** | | | | |
| **Stratified Model** | | | | |
| LM-fixed | 1 | 1.05(0.04) | 0.99 (0.06) | 1.15(0.06) |
| LM-dependent | 1 | 0.89(0.04) | 0.80(0.05) | 0.90(0.06) |
|  | **s/5** | **0.61(0.31)** | **0.50(0.26)** | 1.20(0.31) |
|  | $\left( s/5 \right)^{2}$ | 1.39(0.46) | 0.63(0.18) | 0.88(0.19) |
| **Proportional Hazards** | | | | |
| LM-fixed | 1 | 1.05(0.06) | 0.99(0.06) | 1.15(0.06) |
| LM-dependent | 1 | 0.91(0.04) | 0.81(0.05) | 0.94(0.05) |
|  | s/5 | **0.40(0.21)** | 0.52(0.25) | 0.92(0.22) |
|  | $\left( s/5 \right)^{2}$ | 1.58(0.39) | 0.43(0.16) | 1.01(0.16) |
| **Relapses (**$\boldsymbol{N=2858;}\mathcal{D}\boldsymbol{=564}$**)** | | | | |
| **Stratified Model** | | | | |
| LM-fixed | 1 | 0.95(0.04) | 0.80(0.05) | 0.99(0.03) |
| LM-dependent | 1 | 0.86(0.06) | 0.76(0.06) | 0.97(0.05) |
|  | **s/5** | **0.11(0.31)** | **-0.21(0.30)** | **-0.38(0.28)** |
|  | $\left( s/5 \right)^{2}$ | 0.42 (0.11) | 0.54(0.10) | 0.80(0.11) |
| **Proportional Hazards** | | | | |
| LM-fixed | 1 | 0.95(0.04) | 0.80(0.05) | 0.99(0.05) |
| LM-dependent | 1 | 0.86(0.05) | 0.76(0.06) | 0.97(0.05) |
|  | **s/5** | **0.11(0.27)** | **-0.21(0.29)** | **-0.36(0.27)** |
|  | $\left( s/5 \right)^{2}$ | 0.43(0.11) | 0.54(0.11) | 0.77(0.11) |
| **Relapses and/or Worsening of disease (**$\boldsymbol{N=2858;}\mathcal{D}\boldsymbol{=1377}$**)** | | | | |
| **Stratified Model** | | | | |
| LM-fixed | 1 | 0.93(0.04) | 0.70(0.04) | 0.97(0.03) |
| LM-dependent | 1 | 0.77(0.05) | 0.67(0.05) | 0.87(0.04) |
|  | **s/5** | **0.71(0.42)** | **-0.36(0.35)** | **0.21(0.40)** |
|  | $\left( s/5 \right)^{2}$ | 0.66(0.13) | 0.97(0.14) | 0.76(0.12) |
| **Proportional Hazards** | | | | |
| LM-fixed | 1 | 0.93(0.04) | 0.70(0.04) | 0.97(0.03) |
| LM-dependent | 1 | 0.78(0.05) | 0.67(0.04) | 0.87(0.04) |
|  | **s/5** | **0.61(0.35)** | **-0.32(0.33)** | **0.21(0.35)** |
|  | $\left( s/5 \right)^{2}$ | 0.78(0.13) | 0.92(0.14) | 0.74(0.12) |
| $\mathcal{D}\boldsymbol{=}$ Number of events**,** $\boldsymbol{N=}$Number of observations.  **NB:** The cross-validation based prognostic indices were allowed to interact with different landmark times. The time function chosen were $f_{1}\left( s \right)\equiv1$, $f_{2}\left( s \right)\equiv\left( s/5 \right)$, and $f_{3}\left( s \right)\equiv\left( s/5 \right)^{2}$, for time-fixed, linear, and quadratic interactions with the landmark time points, respectively. Non-significant estimates are highlighted.  -In the landmark prediction, we analysed data only for individuals who survived up to, and including the landmark time.  **Landmark supermodels**: Cox regression on CEPI only, or GPI only, or CEPI+GPI, performed on 5 different landmark datasets. The average estimate over the 5 landmark time points are shown here.  **Landmark super learner**: A Cox regression model on CEGPI only, after obtaining estimates from CEPI+GPI at each landmark time. | | | | |

**Supplementary Table 6:** Calibration by risk groups: 5- and 10-years survival probabilities for different clinical endpoints based on the International Prognostic Index (IPI). The resulting categories of the IPI are 1=low risk; 2=low intermediate risk; 3=high intermediate risk; and 4=high risks.

|  | | **Worsening of Disease**  **(**$\boldsymbol{N=2858;}\mathcal{D}\boldsymbol{=1011}$**)** | | **Relapse (RRE)**  **(**$\boldsymbol{N}\boldsymbol{=}\boldsymbol{2858}\boldsymbol{;}\mathcal{D}\boldsymbol{=}\boldsymbol{564}$**)** | | **Relapse or Worsening of Disease**  **(**$\boldsymbol{N=2858;}\mathcal{D}\boldsymbol{=1377}$**)** | |
| --- | --- | --- | --- | --- | --- | --- | --- |
| **Prognostic**  **Index** | **IPI** | **5yrs**  **(SE)** | **10yrs**  **(SE)** | **5yrs**  **(SE)** | **10yrs**  **(SE)** | **5yrs**  **(SE)** | **10yrs**  **(SE)** |
| $\mathbf{CEPI}$ | 1. LR | 0.88(0.01) | 0.68(0.04) | 0.98(0.01) | 0.95(0.01) | 0.81(0.01) | 0.57(0.03) |
|  | 2. LIR | 0.55(0.02) | 0.16(0.03) | 0.96(0.01) | 0.87(0.02) | 0.52(0.02) | 0.18(0.03) |
|  | 3. HIR | 0.54(0.02) | 0.15(0.04) | 0.82(0.01) | 0.55(0.03) | 0.38(0.02) | 0.08(0.02) |
|  | 4. HR | 0.10(0.02) | 0.00(0.01) | 0.38(0.02) | 0.05(0.01) | 0.06(0.01) | 0.00(0.01) |
|  |  |  |  |  |  |  |  |
| $\mathbf{GPI}$ | 1. LR | 0.82(0.02) | 0.59(0.04) | 0.97(0.01) | 0.93(0.01) | 0.76(0.02) | 0.52(0.03) |
|  | 2. LIR | 0.60(0.02) | 0.26(0.04) | 0.94(0.01) | 0.85(0.02) | 0.53(0.02) | 0.21(0.03) |
|  | 3. HIR | 0.48(0.02) | 0.14(0.03) | 0.84(0.01) | 0.62(0.03) | 0.40(0.02) | 0.11(0.02) |
|  | 4. HR | 0.35(0.02) | 0.06(0.03) | 0.48(0.02) | 0.49(0.02) | 0.14(0.02) | 0.01(0.01) |
|  |  |  |  |  |  |  |  |
| $\mathbf{CEGPI}$ | 1. LR | 0.86(0.01) | 0.65(0.04) | 0.98(0.01) | 0.95(0.01) | 0.82(0.01) | 0.57(0.03) |
|  | 2. LIR | 0.56(0.02) | 0.16(0.04) | 0.97(0.01) | 0.90(0.02) | 0.51(0.02) | 0.15(0.02) |
|  | 3. HIR | 0.53(0.02) | 0.14(0.03) | 0.81(0.02) | 0.52(0.03) | 0.38(0.02) | 0.07(0.01) |
|  | 4. HR | 0.08(0.03) | 0.00(0.01) | 0.38(0.03) | 0.05(0.01) | 0.05(0.01) | 0.00(0.01) |
| **NB:** The corresponding risk quantiles for the IPIs are: $"\mathbf{IPI 1}"=$ 0-25%; $\text{"}\text{IPI 2}\text{"}=$25-50%; $"\mathbf{IPI 3}"=$50-75% and $"\mathbf{IPI 4}"=$ 75-100%; **LR**: Low risk; **LIR**: Low intermediate risk; **HIR**: High intermediate risk; **HR**: High risk; $\mathbf{CPI}$, $\mathbf{GPI}$ and $\mathbf{CGPI}$ are the cross-validated clinical, genetic, and clinico-genotypic prognostic indices respectively.  **CEPI**: Clinical-Env Prognostic Index (Clinical ǂ Environmental predictors)  **GPI:** Genetic Prognostic Index (Cumulative effects of single nucleotide polymorphisms markers)  **CEGPI**: Clinical-Env-Genotypic Prognostic Index = CEPI + GPI (clinical + environmental + genetic) | | | | | | | |
